# DNA methylation and gene expression integration in cardiovascular disease

**DOI:** 10.1186/s13148-021-01064-y

**Published:** 2021-04-09

**Authors:** Guillermo Palou-Márquez, Isaac Subirana, Lara Nonell, Alba Fernández-Sanlés, Roberto Elosua

**Affiliations:** 1 Cardiovascular Epidemiology and Genetics Research Group, Hospital del Mar Medical Research Institute (IMIM), Dr Aiguader 88, 08003 Barcelona, Spain; 2 Pompeu Fabra University (UPF), Barcelona, Spain; 3 Institute for Research in Biomedicine (IRB Barcelona), The Barcelona Institute of Science and Technology, Barcelona, Spain; 4 CIBER Epidemiology and Public Health (CIBERESP), Barcelona, Spain; 5 MARGenomics, Hospital del Mar Medical Research Institute (IMIM), Barcelona, Spain; 6 MRC Integrative Epidemiology Unit at the University of Bristol, Bristol, UK; 7 CIBER Cardiovascular Diseases (CIBERCV), Barcelona, Spain; 8 Medicine Department, Faculty of Medicine, University of Vic-Central University of Catalonia (UVic-UCC), Vic, Spain

**Keywords:** DNA methylation, Gene expression, Multi-omics integration, Cardiovascular disease, MOFA, Unsupervised integration

## Abstract

**Background:**

The integration of different layers of omics information is an opportunity to tackle the complexity of cardiovascular diseases (CVD) and to identify new predictive biomarkers and potential therapeutic targets. Our aim was to integrate DNA methylation and gene expression data in an effort to identify biomarkers related to cardiovascular disease risk in a community-based population. We accessed data from the Framingham Offspring Study, a cohort study with data on DNA methylation (Infinium HumanMethylation450 BeadChip; Illumina) and gene expression (Human Exon 1.0 ST Array; Affymetrix). Using the MOFA2 R package, we integrated these data to identify biomarkers related to the risk of presenting a cardiovascular event.

**Results:**

Four independent latent factors (9, 19, 21—only in women—and 27), driven by DNA methylation, were associated with cardiovascular disease independently of classical risk factors and cell-type counts. In a sensitivity analysis, we also identified factor 21 as associated with CVD in women. Factors 9, 21 and 27 were also associated with coronary heart disease risk. Moreover, in a replication effort in an independent study three of the genes included in factor 27 were also present in a factor identified to be associated with myocardial infarction (*CDC42BPB*, *MAN2A2* and *RPTOR*). Factor 9 was related to age and cell-type proportions; factor 19 was related to age and B cells count; factor 21 pointed to human immunodeficiency virus infection-related pathways and inflammation; and factor 27 was related to lifestyle factors such as alcohol consumption, smoking and body mass index. Inclusion of factor 21 (only in women) improved the discriminative and reclassification capacity of the Framingham classical risk function and factor 27 improved its discrimination.

**Conclusions:**

Unsupervised multi-omics data integration methods have the potential to provide insights into the pathogenesis of cardiovascular diseases. We identified four independent factors (one only in women) pointing to inflammation, endothelium homeostasis, visceral fat, cardiac remodeling and lifestyles as key players in the determination of cardiovascular risk. Moreover, two of these factors improved the predictive capacity of a classical risk function.

**Supplementary Information:**

The online version contains supplementary material available at 10.1186/s13148-021-01064-y.

## Background

Cardiovascular diseases (CVD) are the leading cause of mortality and disease burden worldwide [1, 2] and comprise several diseases with different etiologies that affect the heart or blood vessels. CVD prevention, one of the main public health challenges, is based on population and individual interventions [3]. The former includes strategies affecting the whole population, such as smoking ban policies, whereas individual interventions are tailored to each patient based on the estimation of cardiovascular risk. Cardiovascular risk functions are the most common tool to assess cardiovascular risk. Several functions have been developed and validated; however, their sensitivity is low, as a significant number of CVD events occur in individuals with a low or moderate 10-year risk [4]. Therefore, it is necessary to identify and evaluate new predictive biomarkers to improve cardiovascular risk estimation. Moreover, despite pharmacological success in reducing cardiovascular morbidity and mortality, the search for new pathogenic pathways and therapeutic targets is important because residual cardiovascular risk remains a major concern [5].

CVD comprises complex heterogeneous diseases, resulting from an interplay between omic, physiological, environmental and lifestyle factors. Atherosclerosis is the main common pathogenic mechanism, and individual omic analyses have identified markers associated with atherosclerotic CVD. For instance, genome-wide association studies have identified more than 150 loci related to coronary heart disease (CHD) [6], and epigenome-wide association studies (EWAS) have identified several CpGs showing differential methylation related to CVD risk [7–9]. DNA methylation is one of the mechanisms regulating gene expression, which could also determine CVD risk [10]. However, none of the omic layers of biological information (e.g., genomic, epigenomic, transcriptomic, proteomic, metabolomic) captures the full complexity of CVD.

The integration of different layers of omics information is an opportunity to tackle the complexity of CVD and to identify new predictive biomarkers and potential therapeutic targets [11]. Although this integrative analysis remains challenging because of inherent data-type differences, the field is growing and several methods have already been implemented [12]. These methods can be classified as supervised and unsupervised. The aim of supervised methods is to predict one or more conditions related to a sample, although overfitting may be a concern. In contrast, unsupervised methods explore the data by analyzing the correlations among samples in order to condense or simplify the large volume of data in a reduced number of factors that in turn could be associated with clinical traits. One of these unsupervised methods is multi-omics factor analysis (MOFA) [13, 14].

The aim of this study was to integrate DNA methylation and gene expression data to identify biomarkers related to the risk of presenting a cardiovascular event in the Framingham Offspring Study (FOS) using an unsupervised method.

## Results

### Quality control of DNA methylation and gene expression datasets

From 485,577 CpGs and 2620 samples, 411,019 CpGs and 2055 samples remained after the quality control of the DNA methylation data and the application of inclusion and exclusion criteria (Additional file 2: Fig. S1). From 22,011 transcripts and 1,200 samples, 19,904 transcripts and 914 samples were considered for analysis after the quality control of the gene expression data and the application of inclusion and exclusion criteria (Additional file 2: Fig. S2). In this process, we removed all individuals from the transcriptomic batch 15 in both omic datasets (24 samples in transcriptomics and 25 samples in DNA methylation), as this batch showed a differentiated clustering pattern from the rest of the samples.

The main sociodemographic and clinical characteristics of the analyzed individuals are shown in Table 1. Their characteristics were similar to individuals not included in the analysis.

**Table 1:** Descriptive characteristics of the Framingham Offspring Study participants included in this integration analysis

Variable	Methylation	Gene expression
	*n* = 2055	*n* = 914
Age^a^	65.23 (8.59)	64.48 (8.43)
Sex, male, *n* (%)	871 (42.38)	336 (36.76)
Total cholesterol, mg/dL^a^	190.16 (35.79)	192.30 (35.62)
HDL-C, mg/dL^a,c^	58.91 (18.45)	59.51 (17.96)
Triglycerides, mg/dL^b,c^	100 (73,138)	101 (73,140.75)
SBP, mmHg^a,c^	125.19 (16.83)	125.35 (16.97)
DBP, mmHg^a,c^	72.31 (9.90)	72.65 (10.16)
Glucose, mg/dL^b^	101 (94,109)	100 (93,108)
Smokers, *n* (%)	199 (9.68)	96 (10.50)
BMI, kg/m^2^^a,c^	27.99 (5.30)	27.88 (5.33)
Waist, cm^a,c^	100.56 (14.47)	100.08 (14.67)
CHD, *n* (%)	83 (4.04)	28 (3.06)
CVD, *n* (%)	201 (9.78)	79 (8.64)

*SBP* systolic blood pressure, *DBP* diastolic blood pressure, *BMI* body mass index, *Waist* waist circumference, *CHD* coronary heart disease, *CVD* cardiovascular disease

^a^Mean (standard deviation)

^b^Median (interquartile range)

^c^HDL-C, high-density lipoprotein cholesterol

### Identification of MOFA factors related to CVD using an omics integration approach: main analysis

**Fig. 1:**
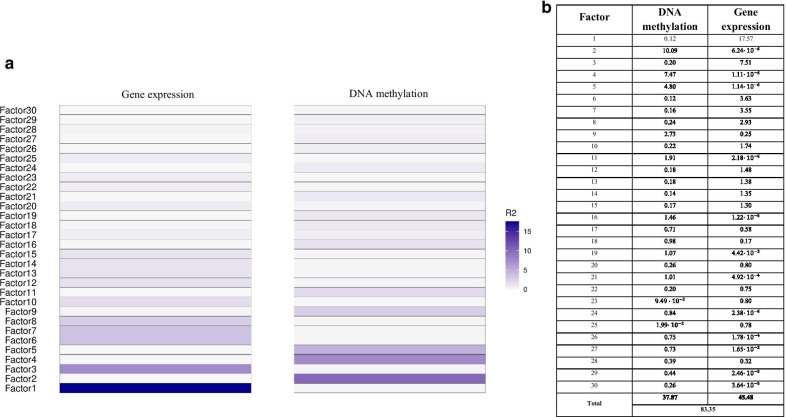
Variance (R^2^) explained by each omic in each factor. **a** Variance explained in a blue-tone color scale. **b** Absolute percentage values of variance explained by each omic in each factor and the total variance explained by all 30 factors

We used the MOFA2 R package to integrate the omics data and identify factors related to the CVD. The 30 identified factors explained 83.35% of the variance of both omics, 45.48% explained by gene expression and 37.87% by DNA methylation (Fig. 1). Surprisingly, most of the factors were mainly explained by only one of the two integrated omics. Correlation coefficients among factors were < 0.20 (Additional file 2: Fig. S3).

### Association between the identified MOFA factors and CVD incidence

**Fig. 2:**
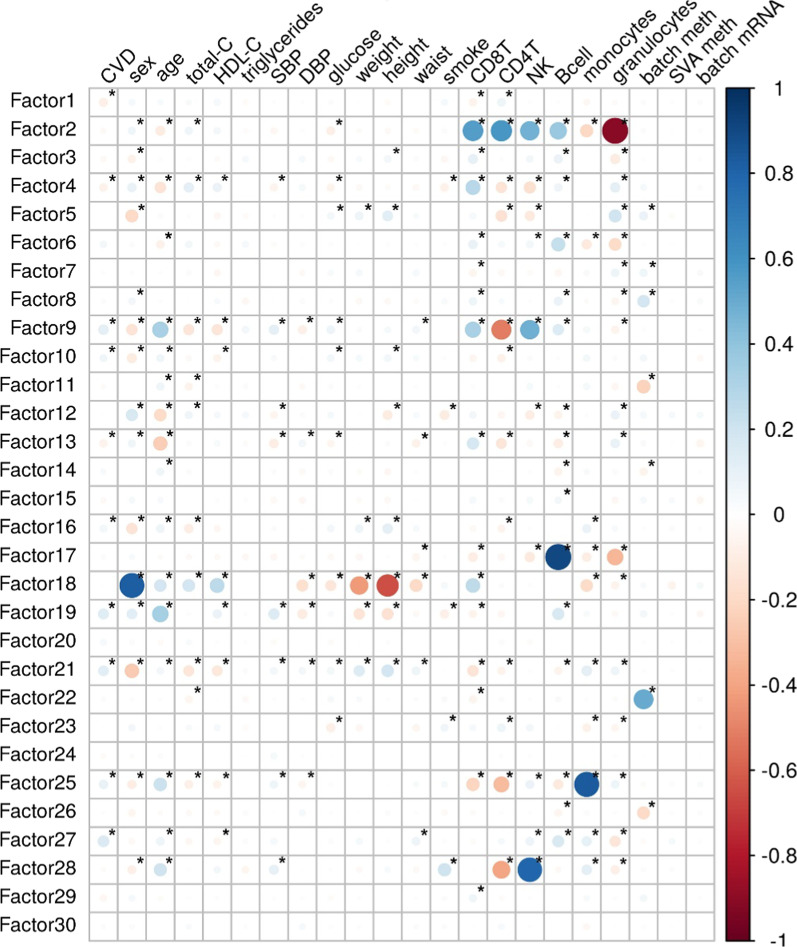
Correlation between the MOFA factors, cardiovascular disease (CVD) incidence and the covariates. Correlation coefficients are represented in a color scale from red, for negative correlations, to blue, for positive correlations. *Statistically significant correlation coefficients

**Fig. 3:**
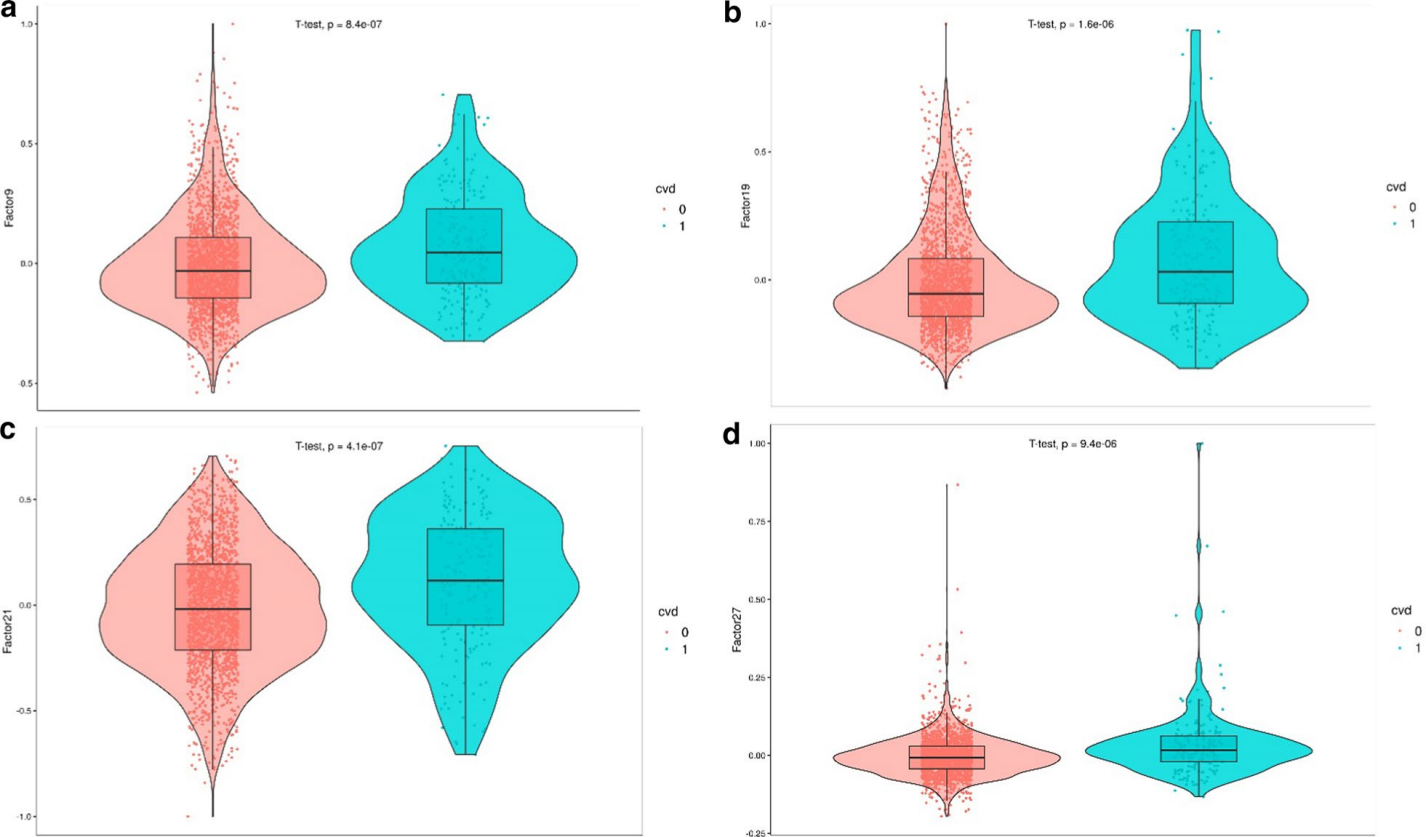
Violin plots of the four factors significantly associated with cardiovascular disease (CVD) incidence in the bivariate analyses: **a** factor 9, **b** factor 19, **c** factor 21 and **d** factor 27. The red-colored group represents individuals not presenting with a CVD event, while the blue-colored group represents those who had a CVD event

The median follow-up of the population was 7.7 years. We first assessed the correlations between the 30 MOFA factors, the main covariates and CVD incidence (Fig. 2). The 30 MOFA factor violin plots stratified by CVD are shown in Additional file 2: Fig. S4. In the main univariate analysis, four factors [9, 19, 21, 27] were associated with CVD risk (Table 2 and Fig. 3). These factors were mostly driven by DNA methylation (Fig. 1). The associations between the four factors and covariates are shown in Additional file 1: Table S1. Factor 9 was mainly related to age, CD4 + T, CD8 + T and NK cells; factor 19 to age and B cells; factor 21 to sex; and factor 27 to B cells.

**Table 2:** Association of the MOFA factors and cardiovascular disease risk (Cox regression): Model 1, adjusted for cell-type counts and one surrogate variable; Model 2, additionally adjusted for age and sex; Model 3, additionally adjusted for total cholesterol, HDL-C levels, glucose, smoking status and systolic and diastolic blood pressure

Model	Association with CVD incidence			Predictive capacity of the Framingham CVR function				
	HR^a^ (95% CI^a^)	*p*-value	FDR correction	C-statistic classical function	C-statistic classical function + factor	*p*-value_C_^a^	NRI^a^ (95% CI)	Clinical NRI^a^ (95% CI^a^)
F9^a^—Model 1	2.05 (1.69, 2.48)	3.04 × 10^−13^	9.44 × 10^−12^	–	–	–	–	–
F9—Model 2	1.56 (1.26, 1.93)	3.48 × 10^−5^	2.7 × 10^−4^	–	–	–	–	–
F9—Model 3	1.42 (1.15, 1.77)	1.37 × 10^−3^	8.46 × 10^−3^	0.73	0.73	0.97	− 2.12 (− 8.17, 3.89)	− 2.38 (− 10.74, 5.98)
F19^a^—Model 1	1.42 (1.26, 1.61)	9.10 × 10^−9^	7.05 × 10^−8^	–	–	–	–	–
F19—Model 2	1.21 (1.06, 1.38)	4.61 × 10^−3^	2.38 × 10^−2^	–	–	–	–	–
F19—Model 3	1.20 (1.05, 1.37)	9 × 10^−3^	4.65 × 10^−2^	0.73	0.74	0.20	0.21 (− 8.01, 8.01)	1.85 (− 8.83, 12.54)
F21^a^ M^a^—Model 1	1.24 (1.02, 1.51)	3.22 × 10^−2^	6.65 × 10^−2^	–	–	–	–	–
F21 M—Model 2	1.21 (1.00, 1.48)	5.56 × 10^−2^	0.13	–	–	–	–	–
F21 M—Model 3	1.21 (0.99, 1.48)	6.38 × 10^−2^	0.15	0.71	0.72	0.30	2.92 (− 8.03, 13.49)	6.61 (− 9.53, 22.76)
F21 W^a^—Model 1	1.81 (1.44, 2.29)	5.52 × 10^−7^	3.42 × 10^−6^	–	–	–	–	–
F21 W—Model 2	1.71 (1.36, 2.15)	5.54 × 10^−6^	5.72 × 10^−5^	–	–	–	–	–
F21 W—Model 3	1.77 (1.39, 2.24)	2.40 × 10^−6^	3.72 × 10^−5^	0.75	0.79	0.01	20.85 (5.04, 37.38)	24.00 (4.55, 43.43)
F27^a^—Model 1	1.38 (1.25, 1.53)	5.98 × 10^−10^	9.28 × 10^−9^	–	–	–	–	–
F27—Model 2	1.38 (1.25, 1.54)	4.48 × 10^−10^	1.39 × 10^−8^	–	–	–	–	–
F27—Model 3	1.36 (1.22, 1.51)	1.08 × 10^−8^	3.35 × 10^−7^	0.73	0.75	0.01	1.23 (− 6.48, 8.44)	4.90 (− 4.37, 14.17)

Cell-type counts and one surrogate variable were used as covariates in the three models. Factor 21 was stratified by sex, as the interaction between this factor and sex was statistically significant. The predictive added-value of each factor when included in the Framingham risk function is also shown in terms of discrimination improvement (C-statistic) and reclassification (Net Reclassification Improvement)

^a^CVD, cardiovascular disease; HR, hazard ratio; CI, confidence interval; *p*-value_C_, *p*-value of the c-statistic comparison; NRI, net reclassification improvement; F9, factor 9; F19, factor 19; F21, factor 21; F27, factor 27; M, men; W, women

In the main multivariate analyses, factors 9, 19 and 27 were associated with CVD independently of classical risk factors. We also found an interaction between factor 21 and sex on CVD risk (*p*-value = 0.007 on model 3); therefore, the analyses were additionally stratified by sex. This factor was associated with CVD only in women.

As expected, most of the CpGs included in the analyses had weight values close to zero in the factors 9, 19, 21 and 27, whereas a few CpGs showed large absolute values, indicating a strong association with the factors (Additional file 2: Fig. S5). We identified the 30 CpGs with the highest weights in those factors (Additional file 2: Fig. S6). The correlation coefficients among the CpGs for each factor are shown in Additional file 2: Fig. S7.

Out of the selected 30 CpGs of each factor, 29, 14, 17 and 13 CpGs of factors 9, 19, 21 and 27, respectively, showed an association with CVD (nominal FDR *p*-value < 0.01, Additional file 1: Tables S2–S5) in the multivariate analysis adjusted for cell-type proportions and one surrogate variable.

### Evaluation of the clinical relevance of the CVD-related factors

We then evaluated the predictive value of including the significant factors in the Framingham risk function (Table 2). The inclusion of factors 21 (only in women) and 27 improved the capacity to discriminate CVD events in the FOS cohort. Reclassification improvement was observed for factor 21 in women, both in the whole group of women and in those with intermediate risk (clinical reclassification).

### Sensitivity analyses and replication of the top features from the CVD-related factors in an independent study

We performed a sensitivity analysis in which we selected the 20,000 CpGs showing the highest variability instead of the most significantly associated with CVD (main analyses). MOFA identified one factor independently associated with CVD. This factor was similar to factor 21 from the main analyses and included CpGs associated with HIV infection pathways, as well as cg06642177, which has been previously related to myocardial infarction.

As a different sensitivity analysis, we also assessed the association of the four identified factors with CHD and found that factors 9, 21 (in women) and 27 showed a similar effect size of association with the two outcomes (Additional file 1: Table S6).

The independent replication was conducted in a case–control study of 391 individuals of the REGICOR –REgistre GIroní del COR-study (196 cases and 195 controls), in which 811,610 CpGs were available after the quality control. In this study, we identified 30 MOFA factors and 10 were associated with myocardial infarction; one of them included three genes that were also included in the factor 27 of the FOS cohort: *CDC42BPB*, *MAN2A2*, and *RPTOR* (Additional file 1: Table S5). None of the top 30 CpGs from factors 9, 19, 21 and 27 were replicated in the REGICOR population (Additional file 1: Tables S7–S10).

## Discussion

We used an unsupervised machine-learning method (MOFA) to identify latent factors that capture biological and technical sources of variability in DNA methylation and gene expression datasets. By integrating these omic data, we identified three factors, almost exclusively explained by DNA methylation, that were independently associated with CVD: factor 19, which included CpGs previously related to age; factor 21 (only in women), which included CpGs previously related to HIV infection pathways and myocardial infarction; and factor 27, which included CpGs previously related to lifestyle factors. Moreover, we report that the inclusion of factor 21 (in women) and factor 27 in the classical Framingham risk function improved its predictive capacity by increasing the discrimination or reclassification.

### Omics integration

The integration of several omics allows modeling data to disentangle the molecular architecture and biological processes of complex traits. Several methods have been proposed for the integration of omic data [15], including MOFA. This method has several advantages, such as identifying latent factors that explain the variability across one or several types of omic data, and the inclusion of samples with missing data in one of the analyzed omic datasets. Among its limitations, as an unsupervised method, are its use of exploratory data analysis to generate hypotheses, the challenge of achieving consistent results and overfitting of the results, although the results seem to be robust in large samples [16].

The added value of data integration was not clearly evidenced in this study, as the identified factors associated with CVD were almost exclusively driven by DNA methylation. However, MOFA is also useful to detect features related to a single omic and latent factors can give more insights into the etiology of CVD, as they offer an integrated understanding and synthesis of the CVD-related molecular pathways and incorporates complex interrelationships across CpGs. This approach could prove to be more useful than the analysis of individual methylation markers.

We aimed to homogenize the number of epigenome and transcriptome data points to be included in the MOFA analysis. As gene expression data included 22,011 transcripts and all of them were included in the main MOFA2 analysis, we selected methylation data to include 20,000 CpGs of the original 411,019. Two main strategies could be used to select 20,000 CpG: either select them based on their variability or based on their association with the outcome of interest (CVD). We selected the latter to enrich our initial dataset with marks showing association with CVD. However, this approach enriches methylation data but not transcriptomic data, and it could explain why the factors associated with CVD only included DNA methylation attributes. Therefore, we conducted a sensitivity analysis based on the CpG variability selection criteria, which identified one MOFA factor independently associated with CVD. This factor only included DNA methylation attributes and was similar to factor 21.

### Identified molecular markers: biological pathways

In this study, we identified four factors related to CVD: 9, 19, 21 (in women) and 27. In a sensitivity analysis focusing on CHD, we found that three of the identified factors were also related to CHD with similar effect sizes to those found in the main analysis with CVD: factors 9, 21 (in women) and 27. Factor 19 was not related to CHD but its association with CVD was marginally significant (HR = 1.20, FDR *p*-value = 0.047). The consistency between analyses points to atherosclerosis-related pathways.

MOFA, as an unsupervised method, only considers methylation and transcriptomic variability, so the identification of the latent factors does not account for covariates. Therefore, some latent factors could reflect variability in cell-type counts in blood, without changes in the molecular characteristics in any of the mature cells of the blood. This phenomena is called polycreodism [17], which in this study is particularly important to account for since cell-type differences could reflect immune-related inflammation, a well-known pathogenic mechanism of atherosclerosis. Thus, the association between MOFA factors and CVD was adjusted for blood cell-type counts to mitigate their potential confounder effect.

Factor 9 was related to age and cell-type proportions. Some of the genes included in this factor have been previously related to cardioprotective effects: *SLC1A5, SLP1* [18, 19]*.* However, the association with CVD was independent of age and cell types. Other genes clustered in this factor are *GALNT2* that shows differential methylation associated with CHD [20], and *PTP4A2* and *JAZF1* that have been related to angiogenesis [21, 22].

Among the genes showing differential methylation features and included in factor 19, we can highlight *MCF2L*, *ZBTB46*, *ANGPTL2*, and *BICD2*. *G*enetic variants in *MCF2L* and *ZBTB46* have been reported to be significantly associated with CHD [23]. ANGPTL2 maintains vascular endothelium homeostasis, having a role in angiogenesis, tissue repair, obesity and atherosclerotic diseases [24]. Finally, genetic variants in *BICD2* have been associated with visceral fat [25]. In summary, this factor suggests several biological factors (inflammation, endothelial homeostasis, visceral fat accumulation) that could explain the association with higher CVD risk.

Factor 21 was associated with CVD exclusively in women. Interestingly, this factor was also observed in the MOFA sensitivity analysis based on the CpG variability selection criteria. Moreover, 16 of the 30 top attributes included in factor 21 were also associated with CHD in the Framingham dataset in a previous integration effort using genomic and epigenomic data and a Random Forest classification model [26]. Twenty-nine out of 30 CpGs from factor 21 have been associated with HIV infection-related pathways [27]. Among the genes showing differential methylation features and included in factor 21, we can highlight *NLRC4*, *NCL*, *PTEN*, *ATM*, and *SGK1*. *NLRC4* and *NCL* contain genetic variants associated with inflammation biomarkers [28, 29]. Genetic variants in *PTEN* and *ATM* genes have been associated with eosinophil count [30] and CHD [31], respectively. Finally, differential methylation in cg06642177 linked to *SKG1* has been previously associated with myocardial infarction [32]. This gene has been considered an important factor in the regulation of inflammation in CVD [33] and contributes to cardiac remodeling and development of heart failure [34]. In summary, this factor points to inflammation, cell cycle regulation and cardiac remodeling as key pathways in CVD risk. We do not have a clear explanation for the differential association with CVD between sexes.

Lastly, factor 27 was mainly related with lifestyle factors: alcohol consumption, body mass index and smoking. Interestingly, we replicated a similar factor including three common genes in an independent case–control study applying the MOFA analysis in REGICOR data. These genes (*CDC42BPB*, *MAN2A2*, *RPTOR*) present differential methylation related to alcohol consumption [35], body mass index [36] and smoking [37], respectively. Genetic variability in *MAN2A2* and *RPTOR* has been related to CHD [38] and body mass index and blood pressure [39], respectively. Finally, another interesting gene included in factor 27 is *ABCA2* that reduces low-density lipoprotein receptor expression [40]. In summary, this factor suggests several biological mechanisms that could mediate the relationship between lifestyle factors and CVD risk.

Our analysis did not replicate previous findings from the Framingham heart study in which they reported, in combination with other cohorts, several CpGs or gene expression signatures related to myocardial infarction and CHD [8, 41]. However, our analysis approach using MOFA latent factors differs from those previously used and could explain these differences.

### Identified molecular markers: clinical predictive added-value

Factors 21 (in women) and 27 improved the discriminative capacity of the Framingham risk function to identify individuals who will develop a CVD in the next 10 years. Reclassification improvement was significant in women for factor 21, as well as in the subgroup of women with intermediate risk. These reclassification results should be replicated in an independent prospective sample.

### Strengths and limitations

The main strength of this study is the large sample size and the community-based design, along with its integrative approach to identify molecular markers related to CVD. In addition, the matrix factorization model of MOFA allows data treatment for individuals with missing values for one of the omics. We should consider the presence of population stratification and familiar relatedness and their potential effects in our results [42]. Potential population stratification would be accounted for using the MOFA latent factors (similar to methylation-based principal components) and surrogate variables, reducing the possibility of reporting false positive results [43]. However, we could not account for familiar relatedness in our analyses to minimize its potential impact on our results. Moreover, we are aware of additional limitations of the study: (1) the number of cases is limited, hampering the statistical power of the study; (2) not all the samples with transcriptomic data could be incorporated in the analysis because of a computational memory limitation; (3) the dimensions of the methylation data were reduced to match the dimensions of the available transcriptomics data, to avoid overrepresentation bias in the factors; (4) we did not replicate the complete analysis in an independent cohort as we did not have access to other populations with data of both omics; (5) MOFA modeling assumes linear association; thus, it does not consider nonlinear relationships between features within and across assays [44]; and (6) CVD include several clinical diseases, introducing some heterogeneity in our main outcome, although the main results for factors 9, 21 and 27 are robust when analyzing CHD.

## Conclusions

This study showed the potential of unsupervised integration methods to provide some insights in the pathogenesis of cardiovascular diseases. We identified four independent factors (one only in women) pointing to inflammation, endothelium homeostasis, visceral fat, cardiac remodeling and lifestyles as key players in the determination of cardiovascular risk. Two of these factors improved the predictive capacity of a classical risk function.

## Methods

### Study design and population

The Framingham Offspring Study (FOS) is a prospective community-based cohort study. FOS data were obtained through the database of Genotypes and Phenotypes (dbGAP, http://dbgap.ncbi.nlm.nih.gov; project number #9047). We included the participants in exam 8 with available DNA methylation data (Framingham Offspring Exam 8 DNA Methylation Study, *n* = 2620; dbGaP Study Accession: phs000724.v7.p11) and gene expression data (NHLBI Framingham SABRe CVD, *n* = 1892; dbGaP Study Accession: phs000363.v17.p11). Participants with previous CVD and those with no follow-up data were excluded.

### DNA methylation assessment

DNA extraction and methylation assessment have been previously fully described [45]. Briefly, DNA was extracted from buffy coat using a standardized method (Puregen TM, Gentra Systems). Genome-wide DNA methylation was assessed using the Infinium HumanMethylation450 BeadChip (Illumina, CA, USA), following the Illumina Infinium HD Methylation protocol [46, 47]. This array is based on the bisulfite conversion of 485,577 unmethylated cytosines across the genome.

The quality control protocol excluded cross-reactive probes [48, 49] and CpGs with a beadcount < 3 in at least 5% of the samples and detection *p*-values > 0.05 in at least 1% of the samples. We also excluded the samples with inconsistent methylation-based predicted and reported sex. Quality control was performed using the wateRmelon (v1.22.0) [50] and minfi (v1.24.0) [51] R packages. We also excluded CpGs located on the sexual chromosomes.

Methylation data were normalized using the Dasen method [50], which involves background adjustment of the methylated and unmethylated intensities, followed by between-array normalization and dye bias correction. The potential presence of batch effect was explored in a multi-dimensional scaling (MDS) plot, and if present it was controlled by regressing out the batch variable using ComBat [52].

Methylation status at each CpG site was reported by M-value. M-values above 4 standard deviations from the average in absolute value were excluded from analysis.

Finally, FlowSorted.Blood.450 k R package (v1.16.0) [53] was used to obtain methylation-based estimates of the blood cell-type counts (B Cells, Monocytes, Granulocytes, Natural Killers, CD8 + T cells and CD4 + T cells). The sva R package (v3.26.0) [54] was used to obtain surrogate variables to account for unmeasured technical or biological variability.

### Gene expression assessment

RNA extraction and gene expression profiling have been previously described [41]. In brief, fasting peripheral whole blood samples were collected in PAXgene™ tubes (PreAnalytiX, Hombrechtikon, Switzerland). RNA was isolated and cDNA was obtained according to the manufacturer’s standard protocols. cDNA was hybridized to the Human Exon 1.0 ST Array (Affymetrix, Inc., Santa Clara, CA). This array consists of over 6 million probes grouped in about 1.2 million probesets, targeted to the majority of known exons in the human genome. Only gene-level analysis (transcript clusters with “core” annotations) was conducted, including 22,011 transcripts.

Computational memory limited the analysis to 1,200 individuals, which we randomly selected from the available 1,892. Quality control of the raw data was performed using the oligo R package (v1.42.0) [55]. We visualized the expression data for the analyzed samples, clustered by batch, using boxplots, Normalized Unscaled Standard Error (NUSE) and Relative Log Expression (RLE) plots. We considered as a potential outlier any sample whose median was above 95% or below 5% quantiles from the distribution of medians for each type of plot. A potential outlier observed in at least 2 out of 3 plots was considered a real outlier and removed from the data. Distribution of the red/green intensity ratio ('M') plotted by the average intensity (‘A’)—MA-plots—was also performed. Data were quantile-normalized, log2 transformed, background substracted and summarized by the Robust Multi-array Average method (RMA) [56] implemented in the oligo package. We removed transcripts with an expression value less than 4 in at least as many samples as the smallest experimental group (201 individuals with CVD). Finally, transcripts located on sexual chromosomes were removed. We explored for batch effect using MDS plots, and if present controlled for it by regressing out the batch variable with ComBat [52]. The group of participants with gene expression data was a subset of the DNA methylation set of participants.

### Clinical cardiovascular events and other covariates assessment

The main clinical outcome was incident CVD that included coronary heart disease (angina, myocardial infarction, coronary revascularization and coronary heart disease death) and other cardiovascular events (heart failure, stroke, transient ischemic accident, carotid revascularization, peripheral artery disease and other circulatory problems). The events were adjudicated by the Framingham event committee. Follow-up included exam 8 (baseline visit) to exam 12. Traditional risk factors at the baseline visit (sex, age, total cholesterol, high-density lipoprotein cholesterol [HDL-C], glucose, systolic and diastolic blood pressure [SBP and DBP, respectively] and smoking status) were used as covariates in the Cox regression analyses.

### MOFA models

To perform the integration of both omics, we used the MOFA2 R package (v0.99.5) [13]. MOFA identifies latent factors that capture biological and technical sources of variability in multi-omics datasets. Mathematically, each factor orders cells through a one-dimensional axis centered at zero. The interpretation of factors is analogous to the interpretation of principal components.

The matrix of methylation data was much larger than the gene expression matrix, which could bias the analysis [13]. We followed an EWAS strategy to reduce the number of CpGs to analyze from the methylation data, selecting the 20,000 CpGs with the lowest *p*-value in the association with CVD. As a sensitivity analysis, we also selected the 20,000 CpGs with the highest variability measured by the standard deviation (recommended by MOFA authors). Data, model and training options were left as default, but the “convergence_mode” train argument was set to “slow” and the “num_factors” to 30.

We determined the variance explained per factor in both omics, and the total variance explained by each omic. As a quality control, we estimated the correlation between factors to check whether they captured unique sources of variation.

MOFA is a completely unsupervised machine-learning method, and the covariates and the presence of CVD were not used for model training. The relationship between the presence of CVD, the covariates and the MOFA factors was analyzed a posteriori*.*

### Statistical analysis

First, the association between the identified MOFA factors and CVD incidence was assessed using Cox proportional hazards regression models using survival (v3.1-12) [57] and Hmisc (v4.4-0) [58] R packages. We defined three models for each MOFA factor: non-adjusted, adjusted for sex and age and additionally adjusted for total cholesterol, HDL-C, SBP, DBP, glucose and smoking. Cell-type counts and one surrogate variable were used as covariates in the three models. We also tested the interaction between the MOFA factors and sex on CVD risk.

Second, we assessed the potential added predictive value of including the CVD-associated MOFA factors in the Framingham risk function by estimating the improvement in discrimination (Harrell’s c statistic), applying the rcorr.cens function of the Hmisc R package, and the net reclassification improvement (NRI), using the nricens R package (v1.6) [59]. We defined three risk categories (low, intermediate and high), applying cutoffs according to National Cholesterol Education Panel (NCEP) guidelines for 10-year risk [60]: [0–10]%, [10–20)%, ≥ 20%, respectively). The expected number of events at 5 years in each risk category (thus, [0–5]%, [5–10]%, ≥ 10%) were calculated using Kaplan–Meier estimates. Moreover, we analyzed the NRI in the group of individuals with intermediate CVD risk—i.e., the clinical NRI—and corrected the bias in NRI estimation in this group [61].

### Biological pathways of the CVD-related MOFA factors

Each MOFA factor is defined by several features of the integrated omics (either CpGs or expressed genes). Features with score values close to zero are not related to the factor, whereas features with large absolute values have a strong association with it. The sign of the weight indicates the direction of the association. We identified the features with the highest scores defining the factors related to CVD and, using the corrplot R package (v0.84) [62], estimated the correlation between all the features included in one factor to identify those that captured unique sources of variation. The top 30 CpGs within each factor were checked in the EWAS catalog [63], and we annotated the expressed genes using the Affymetrix HuEx-1_0-st-v2 annotation file. Finally, we assessed the association between each of the top 30 features of each factor and CVD risk using Cox regression models.

### Sensitivity analysis and independent replication of the MOFA factors and the top CpG features related to CVD

As a sensitivity analysis, we examined the association between the identified MOFA factors and CHD, to assess the consistency of the effect sizes of the associations between MOFA factors and CVD, and those with CHD.

Two approaches were used to replicate the main DNA methylation markers identified as relating to CVD in an independent EWAS from the REGICOR study [64]. This study included 208 consecutive myocardial infarction cases (104 women, overrepresented in the study) and 208 age- and sex-matched controls. DNA methylation was assessed with the Illumina HumanMethylationEPIC array, and data quality control was very similar to that performed in the FOS population [64]. Additional information can be found in Additional file 3. First, we ran a new and similar analysis in the replication cohort REGICOR, using the 40,000 CpGs more significantly associated with myocardial infarction in the REGICOR study (those with the lowest *p*-value in the EWAS). Thus, we identified latent factors using the MOFA2 R package and assessed their association with myocardial infarction. Then, we assessed whether the MOFA factors related to CVD (in FOS) and myocardial infarction (in REGICOR) pointed to similar significant biological pathways. Second, we identified the top 30 CpGs that defined the MOFA factors related to CVD in the FOS and assessed for their association with myocardial infarction in the REGICOR study.

## Supplementary Information

**Additional file 1:** Supplementary tables.

**Additional file 2:** Supplementary figures.

**Additional file 3:** Supplementary material and methods.

## Data Availability

The datasets analyzed during the current study are available in the dbGAP repository: https://www.ncbi.nlm.nih.gov/projects/gap/cgi-bin/study.cgi?study_id=phs000007.v31.p12. Specifically, the dbGaP Study Accession codes were phs000724.v7.p11 and phs000363.v17.p11, for the DNA methylation and gene expression data, respectively. The code underlying this article is available at https://github.com/gpalou4/TFM. Supplementary information: Supplementary material is available at https://github.com/gpalou4/TFM/tree/master/manuscript/supp_material

## Abbreviations

CHD: Coronary heart disease
CVD: Cardiovascular diseases
dbGAP: Database of genotypes and phenotypes
DBP: Diastolic blood pressure
EWAS: Epigenome-wide association studies
FDR: False discovery rate
FOS: Framingham offspring study
HDL-C: High-density lipoprotein cholesterol
MOFA: Multi-omics factor analysis
REGICOR: REgistre GIroní del COR
SBP: Systolic blood pressure
