# Supplementary material for: DNA methylation and gene expression integration in cardiovascular disease
Source: Clin Epigenetics. 2021 Apr 9;13:75. doi: 10.1186/s13148-021-01064-y (PMC8034168; doi:10.1186/s13148-021-01064-y)
Supplement: Supplementary file 1 — Additional file 1: Supplementary tables. [file 13148_2021_1064_MOESM1_ESM.docx]

**SUPPLEMENTARY TABLES**

**Table S1.** Correlation coefficients of Factors 9, 19, 21 and 27 with the main covariates and cardiovascular disease incidence.

| **Covariate** | **Factor 9** | **Factor 19** | | **Factor 21** | **Factor 27** |
| --- | --- | --- | --- | --- | --- |
| Age | 0.32 | | 0.34 | 0.06 | 0.08 |
| Sex | -0.14 | | 0.12 | -0.25 | -0.04 |
| Smoker | ${-5.02\cdot10}^{-3}$ | | $-0.09$ | ${9.79\cdot10}^{-3}$ | -0.02 |
| Height | $0.04$ | | -0.16 | 0.18 | $0.02$ |
| Weight | 0.03 | | -0.14 | 0.14 | 0.05 |
| Total cholesterol | -0.13 | | -0.02 | -0.13 | -0.05 |
| HDL-C^*^ | -0.14 | | 0.09 | -0.13 | -0.06 |
| Glucose | 0.08 | | 0.03 | 0.06 | 0.02 |
| Granulocytes | -0.06 | | -0.03 | 0.08 | -0.14 |
| Triglycerides^*^ | 0.05 | | -0.02 | 0.03 | 0.03 |
| SBP^*^ | 0.11 | | 0.14 | 0.06 | 0.05 |
| DBP^*^ | -0.08 | | -0.11 | 0.06 | ${-3.58\cdot10}^{-3}$ |
| CD4+ T cells | -0.51 | | -0.05 | -0.07 | 0.04 |
| CD8+ T cells | 0.32 | | -0.06 | -0.15 | -0.02 |
| NK cells^*^ | 0.48 | | ${-1.73\cdot10}^{-3}$ | 0.01 | 0.08 |
| B cells | 0.15 | | 0.17 | $-0.06$ | 0.16 |
| Monocytes | $0.05$ | | $-0.04$ | 0.12 | 0.11 |
| CVD^*^ | 0.12 | | 0.13 | 0.12 | 0.15 |

*HDL-C, High-density lipoprotein cholesterol; SBP, Systolic blood pressure; DBP, Diastolic blood pressure; NK cells, Natural Killer cells; CVD, Cardiovascular disease

**Table S2. Association of the top CpGs of Factor 9 and cardiovascular disease incidence obtained in the Cox regression analysis adjusted by cell type proportions and one surrogate variable, and annotations among these CpGs and other traits reported in the EWAS catalog.**

| **Feature** | | | **Association with CVD incidence** | | | | **EWAS catalog annotation** | | | | |
| --- | --- | --- | --- | --- | --- | --- | --- | --- | --- | --- | --- |
| *CpG* | *Location* | *Gene* | *HR** | *95% CI** | *P-value* | *FDR correction* | *Trait* | *Beta* | *P-value* | *PMID** |  |
| cg02352281 | chr3:46139305 | *-* | 0.39 | [0.26,0.57] | 1.49·${10}^{-6}$ | 2.98·${10}^{-6}$ | Clear cell renal carcinoma | NA | 1.7 ·${10}^{-36}$ | 23526956 |  |
| cg21884231 | chr3:170303721 | *SLC7A14* | 2.31 | [1.68,3.17] | 2.30·${10}^{-7}$ | 6.28·${10}^{-7}$ | Clear cell renal carcinoma | NA | 9.1 ·${10}^{-28}$ | 23526956 |  |
| cg06577205 | chr5:15500714 | *FBXL7* | 2.23 | [1.52,3.27] | 3.80·${10}^{-5}$ | 5.43·${10}^{-5}$ | Age | NA | 1.1 ·${10}^{-26}$ | 25404168 |  |
| cg00356183 | chr7:751833 | *PRKAR1B* | 1.72 | [1.28,2.32] | 3.42·${10}^{-4}$ | 3.80·${10}^{-4}$ | Pancreatic ductal adenocarcinoma | NA | 7.4 ·${10}^{-10}$ | 24500968 |  |
| cg03044249 | chr7:70597065 | *WBSCR17* | 1.86 | [1.37,2.54] | 8.14·${10}^{-5}$ | 9.76·${10}^{-5}$ | Age group | 0.88 | 1.9 ·${10}^{-14}$ | 25888029 |  |
| cg23130097 | chr8:120220484 | *MAL2* | 2.14 | [1.57,2.92] | 1.78 ·${10}^{-6}$ | 3.34·${10}^{-6}$ | age | 0.03 | 3 ·${10}^{-8}$ | 31426852 |  |
| cg24430034 | chr13:110386176 | *-* | 2.26 | [1.66,3.06] | 1.77·${10}^{-7}$ | 6.16·${10}^{-7}$ | smoking | 5.9·${10}^{-3}$ | 8.5 ·${10}^{-15}$ | 31536415 |  |
| cg03287340 | chr19:50651195 | *-* | 1.45 | [1.06,2.00] | 0.02 | 0.02 | sex | NA | 1.3 ·${10}^{-7}$ | 26500701 |  |
| cg01437204 | chr1:202130344 | *PTPN7* | 0.29 | [0.19,0.45] | 1.80·${10}^{-8}$ | 1.30·${10}^{-7}$ | Age group | -0.98 | 4.5 ·${10}^{-21}$ | 25888029 |  |
| cg13359998 | chr1:230241764 | *GALNT2* | 0.31 | [0.21,0.48] | 8.53·${10}^{-8}$ | 3.66·${10}^{-7}$ | Age group | -0.66 | 6.6 ·${10}^{-18}$ | 25888029 |  |
| cg16401270 | chr1:32404469 | *PTP4A2* | 0.43 | [0.28,0.66] | 1.29·${10}^{-4}$ | 1.49·${10}^{-4}$ | Age group | -0.92 | 2.1 ·${10}^{-22}$ | 25888029 |  |
| cg25375916 | chr3:155570275 | *SLC33A1* | 0.34 | [0.20,0.57] | 4.63·${10}^{-5}$ | 6.03·${10}^{-5}$ | smoking | -6.52·${10}^{-3}$ | 1.3·${10}^{-9}$ | 31536415 |  |
| cg21099332 | chr5:39270715 | *-* | 0.31 | [0.19,0.51] | 4.27·${10}^{-6}$ | 6.75·${10}^{-6}$ | Primary Sjogrens syndrome | Na | 1.9 ·${10}^{-15}$ | 26857698 |  |
| cg01657995 | chr6:31804883 | *C6orf48;SNORD52* | 0.26 | [0.16,0.41] | 8.55·${10}^{-9}$ | 1.28·${10}^{-6}$ | Age group | -0.74 | 5.4 ·${10}^{-16}$ | 25888029 |  |
| cg01702055 | chr6:13303065 | *-* | 0.26 | [0.15,0.43] | 3.41·${10}^{-7}$ | 8.53·${10}^{-7}$ | Gestational age | 1.74·${10}^{-3}$ | 2.7 ·${10}^{-14}$ | 27717397 |  |
| cg02010481 | chr7:28218524 | *JAZF1* | 0.26 | [0.16,0.41] | 2.16·${10}^{-8}$ | 1.30·${10}^{-7}$ | Age group | -0.75 | 6.3 ·${10}^{-23}$ | 25888029 |  |
| cg03183540 | chr8:134931756 | *-* | 0.31 | [0.20,0.49] | 3.96·${10}^{-7}$ | 9.14·${10}^{-7}$ | Age group | -0.78 | 1.3 ·${10}^{-23}$ | 25888029 |  |
| cg09077126 | chr10:72015695 | *NPFFR1* | 2.58 | [1.85,3.59] | 2.10·${10}^{-8}$ | 1.30·${10}^{-3}$ | Age group | 0.77 | 1.1 ·${10}^{-20}$ | 25888029 |  |
| cg04111435 | chr11:63448455 | *RTN3* | 0.25 | [0.16,0.41] | 4.09·${10}^{-8}$ | 2.05·${10}^{-7}$ | Age group | -0.65 | 3 ·${10}^{-14}$ | 25888029 |  |
| cg13984040 | chr12:125258948 | *-* | 0.41 | [0.29,0.57] | 2.22·${10}^{-7}$ | 6.28·${10}^{-7}$ | Age group | -0.85 | 1.5 ·${10}^{-15}$ | 25888029 |  |
| cg15095917 | chr17:7482694 | *CD68* | 0.31 | [0.19,0.51] | 3.67·${10}^{-6}$ | 6.18·${10}^{-6}$ | Age group | -0.55 | 1.8 ·${10}^{-15}$ | 25888029 |  |
| cg23691006 | chr17:1510666 | *SLC43A2* | 0.34 | [0.21,0.55] | 8.67·${10}^{-6}$ | 1.30·${10}^{-5}$ | Age group | -0.74 | 5.5 ·${10}^{-21}$ | 25888029 |  |
| cg01406381 | chr19:47288263 | *SLC1A5* | 0.43 | [0.28,0.65] | 7.40·${10}^{-5}$ | 9.26·${10}^{-5}$ | Age group | -1.23 | 1.9 ·${10}^{-28}$ | 25888029 |  |
| cg21766592 | chr19:47288066 | *SLC1A5* | 0.42 | [0.29,0.61] | 3.71·${10}^{-6}$ | 6.18·${10}^{-6}$ | Smoking | 7.72·${10}^{-3}$ | 6.2 ·${10}^{-20}$ | 31536415 |  |
| cg22540135 | chr19:51898904 | *-* | 0.42 | [0.25,0.71] | 1.30·${10}^{-3}$ | 1.39·${10}^{-3}$ | Age group | -0.76 | 3.2 ·${10}^{-16}$ | 25888029 |  |
| cg07668993 | chr20:43883307 | *SLPI* | 0.31 | [0.20,0.49] | 4.37·${10}^{-7}$ | 9.37·${10}^{-7}$ | Age group | -0.67 | 3.7 ·${10}^{-20}$ | 25888029 |  |
| cg08548498 | chr20:43883546 | *SLPI* | 0.33 | [0.22,0.50] | 1.85·${10}^{-7}$ | 6.16·${10}^{-7}$ | Age group | -0.77 | 1.2 ·${10}^{-15}$ | 25888029 |  |
| cg12966875 | chr20:43883746 | *SLPI* | 0.41 | [0.26,0.63] | 4.42·${10}^{-5}$ | 6.03·${10}^{-5}$ | Age group | -0.68 | 3.2 ·${10}^{-15}$ | 25888029 |  |
| cg19473529 | chr20:48726783 | *UBE2V1;TMEM189-UBE2V1* | 0.43 | [0.25,0.73] | 1.81·${10}^{-3}$ | 1.87·${10}^{-3}$ | age | 6.6·${10}^{-4}$ | 1.5 ·${10}^{-18}$ | 23177740 |  |
| cg23480341 | chr20:43883705 | *SLPI* | 0.32 | [0.22,0.47] | 4.81·${10}^{-9}$ | 1.28·${10}^{-7}$ | Age group | -0.84 | 8.6 ·${10}^{-19}$ | 25888029 |  |

*HR, Hazard Ratio; CI, Confidence Interval of the HR; FDR, False Discovery Rate; PMID, PubMed Identification number; NA, not available*.*

**Table S3. Association of the top CpGs of Factor 19 and cardiovascular disease incidence obtained in the Cox regression analysis adjusted by cell type proportions and one surrogate variable, and annotations among these CpGs and other traits reported in the EWAS catalog.**

| **Feature** | | | **Association with CVD incidence** | | | | **EWAS catalog annotation** | | | | |
| --- | --- | --- | --- | --- | --- | --- | --- | --- | --- | --- | --- |
| *CpG* | *Location* | *Gene* | *HR** | *95% CI** | *P-value* | *FDR correction* | *Trait* | *Beta* | *P-value* | *PMID** |  |
| cg13060114 | chr1:36038950 | *TFAP2E* | 0.96 | [0.74,1.24] | 0.73 | 0.78 | Clear cell renal carcinoma | NA | 8.6 ·${10}^{-27}$ | 23526956 |  |
| cg00084338 | chr6:170595920 | *DLL1* | 0.81 | [0.69,0.96] | 1.42·${10}^{-2}$ | 2.24·${10}^{-2}$ | Sex | NA | 1.7 ·${10}^{-14}$ | 26500701 |  |
| cg22878489 | chr6:33245701 | *B3GALT4* | 0.33 | [0.23,0.49] | 1.06·${10}^{-8}$ | 3.15·${10}^{-7}$ | Sex | NA | 2 ·${10}^{-18}$ | 26500701 |  |
| cg09316140 | chr8:21912474 | *EPB49* | 0.66 | [0.48,0.90] | 9.76·${10}^{-3}$ | 1.63·${10}^{-2}$ | Age | NA | 1.8 ·${10}^{-78}$ | 28056824 |  |
| cg26963277 | chr11:2722407 | *KCNQ1OT1;KCNQ1* | 0.45 | [0.32,0.62] | 2.30·${10}^{-6}$ | 1.38·${10}^{-5}$ | Current vs never smoking | NA | 4.5 ·${10}^{-22}$ | 23691101 |  |
| cg06673684 | chr13:113646815 | *MCF2L* | 1.06 | [0.74,1.50] | 0.76 | 0.79 | Fetal vs adult liver | -1.76 | 7.4 ·${10}^{-33}$ | 25282492 |  |
| cg24146100 | chr13:99737448 | *DOCK9* | 0.52 | [0.37,0.73] | 1.5·${10}^{-4}$ | 6.43·${10}^{-4}$ | Fetal vs adult liver | -3.19 | 6.8 ·${10}^{-51}$ | 25282492 |  |
| cg09479241 | chr17:27052676 | *TLCD1* | 0.61 | [0.47,0.79] | 2.52·${10}^{-4}$ | 9.45·${10}^{-4}$ | Age | NA | 1.6 ·${10}^{-15}$ | 28056824 |  |
| cg14170999 | chr17:79380515 | *BAHCC1* | 1.08 | [0.87,1.33] | 0.49 | 0.54 | Fetal vs adult liver | -1.46 | 1 ·${10}^{-28}$ | 25282492 |  |
| cg04164838 | chr19:14591148 | *GIPC1* | 0.76 | [0.62,0.93] | 7.96·${10}^{-3}$ | 1.49·${10}^{-2}$ | Fetal vs adult liver | -1.82 | 3.8 ·${10}^{-38}$ | 25282492 |  |
| cg06693983 | chr19:55889216 | *TMEM190* | 0.76 | [0.64,0.90] | 1.66·${10}^{-3}$ | 4.16·${10}^{-3}$ | Age | NA | 5.4 ·${10}^{-127}$ | 28056824 |  |
| cg25293806 | chr19:55889387 | *TMEM190* | 0.62 | [0.44,0.89] | 8.23·${10}^{-3}$ | 1.49·${10}^{-2}$ | Age | NA | 1.5 ·${10}^{-112}$ | 28056824 |  |
| cg16737517 | chr20:62406677 | *ZBTB46* | 0.73 | [0.58,0.91] | 5.99·${10}^{-3}$ | 1.28·${10}^{-2}$ | Age | NA | 2.4 ·${10}^{-54}$ | 28056824 |  |
| cg26372517 | chr1:36039159 | *TFAP2E* | 0.85 | [0.61,1.18] | 0.33 | 0.38 | Chronic obstructive pulmonary disease | 0.06 | 8.4 ·${10}^{-05}$ | 27564456 |  |
| cg06868100 | chr7:29606349 | *PRR15* | 0.78 | [0.61,0.99] | 3.77·${10}^{-2}$ | 4.92·${10}^{-2}$ | Sex | NA | 0 ·${10}^{-00}$ | 26500701 |  |
| cg08952306 | chr7:101962123 | *SH2B2* | 0.74 | [0.56,0.98] | 3.46·${10}^{-2}$ | 4.71·${10}^{-2}$ | Age | NA | 3.3 ·${10}^{-64}$ | 28056824 |  |
| cg09423312 | chr7:1163549 | *C7orf50* | 0.63 | [0.48,0.82] | 6.29·${10}^{-4}$ | 1.89·${10}^{-3}$ | Alcohol consumption | -2.11 ·${10}^{-4}$ | 7.4 ·${10}^{-33}$ | 27843151 |  |
| cg11908057 | chr7:27171154 | *HOXA4* | 0.63 | [0.48,0.84] | 1.48·${10}^{-3}$ | 4.03·${10}^{-3}$ | Age | NA | 7.4 ·${10}^{-117}$ | 28056824 |  |
| cg23363754 | chr7:29606082 | *PRR15* | 0.80 | [0.65,0.98] | 2.85·${10}^{-2}$ | 4.27·${10}^{-2}$ | Sex | NA | 0 ·${10}^{-00}$ | 26500701 |  |
| cg01028796 | chr9:95525261 | *BICD2* | 0.42 | [0.30,0.60] | 1.62·${10}^{-6}$ | 1.21·${10}^{-5}$ | Age | -0.02 | 8.3 ·${10}^{-17}$ | 28811542 |  |
| cg14281592 | chr9:129884313 | *ANGPTL2;*  *RALGPS1* | 0.58 | [0.43,0.79] | 6.13·${10}^{-4}$ | 1.89·${10}^{-3}$ | Age | NA | 9.6 ·${10}^{-110}$ | 28056824 |  |
| cg03494429 | chr12:4417093 | *-* | 1.28 | [1.02,1.59] | 3.12·${10}^{-2}$ | 4.46·${10}^{-2}$ | Age | NA | 9.1 ·${10}^{-102}$ | 28056824 |  |
| cg02513379 | chr16:27414281 | *IL21R* | 0.37 | [0.26,0.53] | 7.12·${10}^{-8}$ | 7.12·${10}^{-7}$ | Age | NA | 9.5 ·${10}^{-31}$ | 28056824 |  |
| cg07839457 | chr16:57023022 | *NLRC5* | 0.49 | [0.39,0.63] | 2.10·${10}^{-8}$ | 3.15·${10}^{-7}$ | HIV infection | -3.52 | 1.5 ·${10}^{-22}$ | 27105112 |  |
| cg01695406 | chr19:55889276 | *TMEM190* | 0.70 | [0.54,0.91] | 8.42·${10}^{-3}$ | 1.49·${10}^{-3}$ | Age | NA | 7.6 ·${10}^{-126}$ | 28056824 |  |
| cg10064322 | chr19:14591203 | *GIPC1* | 0.50 | [0.32,0.77] | 2.04·${10}^{-3}$ | 4.7·${10}^{-3}$ | Age | NA | 1.1 ·${10}^{-94}$ | 28056824 |  |
| cg19492423 | chr19:18700933 | *C19orf60* | 0.77 | [0.60,0.99] | 4.21·${10}^{-2}$ | 5.26·${10}^{-2}$ | Age | NA | 4.7 ·${10}^{-67}$ | 28056824 |  |
| cg25979108 | chr19:18700553 | *C19orf60* | 0.76 | [0.54,1.07] | 0.11 | 0.13 | Clear cell renal carcinoma | NA | 5.8 ·${10}^{-36}$ | 23526956 |  |
| cg01735357 | chr20:61732467 | *HAR1B;HAR1A* | 0.97 | [0.72,1.31] | 0.84 | 0.84 | Age | NA | 2.9 ·${10}^{-60}$ | 28056824 |  |
| cg01127300 | chr22:38614796 | *-* | 0.45 | [0.31,0.66] | 3.71·${10}^{-5}$ | 1.9·${10}^{-4}$ | Age | NA | 1.8 ·${10}^{-120}$ | 28056824 |  |

*HR, Hazard Ratio; CI, Confidence Interval of the HR; FDR, False Discovery Rate; PMID, PubMed Identification number; NA, not available*.*

**Table S4. Association of the top CpGs of Factor 21 and cardiovascular disease incidence obtained in the Cox regression analysis adjusted by cell type proportions and one surrogate variable, and annotations among these CpGs and other traits reported in the EWAS catalog.**

| **Feature** | | | | **Association with CVD incidence** | | | | | **EWAS catalog annotation** | | | | |
| --- | --- | --- | --- | --- | --- | --- | --- | --- | --- | --- | --- | --- | --- |
| *CpG* | *Location* | *Gene* | *HR** | | *95% CI** | *P-value* | *FDR correction* | *Trait* | | *Beta* | *P-value* | *PMID** |  |
| cg02994863 | chr1:64059297 | *PGM1* | 2.19 | | [1.59,3.01] | 1.26·${10}^{-6}$ | 2.94·${10}^{-6}$ | HIV infection | | -0.53 | 1.30·${10}^{-6}$ | 27105112 |  |
| cg04364194 | chr1:202114085 | *ARL8A* | 2.44 | | [1.76,3.39] | 1.04·${10}^{-7}$ | 5.89·${10}^{-7}$ | HIV infection | | -0.32 | 1.50·${10}^{-6}$ | 27105112 |  |
| cg07959491 | chr1:204347150 | *-* | 1.05 | | [0.80,1.38] | 0.72 | 0.82 | HIV infection | | 3.9 | 6.1·${10}^{-14}$ | 27105112 |  |
| cg16304914 | chr1:44173101 | *ST3GAL3* | 1.07 | | [0.77,1.48] | 0.70 | 0.82 | HIV infection | | 1.7 | 5.7·${10}^{-12}$ | 27105112 |  |
| cg24609304 | chr2:32483287 | *NLRC4* | 1.16 | | [0.88,1.53] | 0.30 | 0.39 | HIV infection | | -3.70 | 1.8·${10}^{-17}$ | 27105112 |  |
| cg26258108 | chr2:232329189 | *NCL* | 2.90 | | [1.93,4.35] | 3.03·${10}^{-7}$ | 8.26·${10}^{-7}$ | HIV infection | | -0.29 | 6.7·${10}^{-7}$ | 27105112 |  |
| cg10341991 | chr3:48229869 | *CDC25A* | 3.29 | | [2.03,5.32] | 1.27·${10}^{-6}$ | 2.94·${10}^{-6}$ | HIV infection | | -0.67 | 1.6·${10}^{-7}$ | 27105112 |  |
| cg06642177 | chr6:13449634 | *SGK1* | 1.81 | | [1.45,2.26] | 1.36·${10}^{-7}$ | 5.89·${10}^{-7}$ | Myocardial infarction | | 0.02 | 4.3·${10}^{-8}$ | 28515798 |  |
| cg15062310 | chr7:100450120 | *SLC12A9* | 2.26 | | [1.68,3.04] | 6.29·${10}^{-8}$ | 75.89·${10}^{-7}$ | HIV infection | | -0.77 | 2·${10}^{-6}$ | 27105112 |  |
| cg25183890 | chr8:94929275 | *PDP1* | 2.46 | | [1.49,4.04] | 4.08·${10}^{-4}$ | 7.65·${10}^{-4}$ | HIV infection | | -0.21 | 2.2·${10}^{-05}$ | 27105112 |  |
| cg10041390 | chr10:89623018 | *PTEN;KILLIN* | 2.53 | | [1.85,3.46] | 5.63·${10}^{-9}$ | 1.69·${10}^{-7}$ | HIV infection | | -0.87 | 2.1·${10}^{-7}$ | 27105112 |  |
| cg06750635 | chr11:108093419 | *ATM;NPAT* | 1.16 | | [0.8,1.68] | 0.45 | 0.56 | HIV infection | | 2.2 | 9·${10}^{-14}$ | 27105112 |  |
| cg07790826 | chr11:70049435 | *FADD* | 0.98 | | [0.77,1.24] | 0.86 | 0.86 | HIV infection | | 2.8 | 5.1·${10}^{-15}$ | 27105112 |  |
| cg04509559 | chr14:69864994 | *ERH;SLC39A9* | 1.05 | | [0.71,1.57] | 0.80 | 0.83 | HIV infection | | -1.83 | 1.4·${10}^{-11}$ | 27105112 |  |
| cg14860120 | chr15:48470606 | *MYEF2* | 1.38 | | [0.93,2.04] | 0.11 | 0.16 | HIV infection | | 1.5 | 7.2·${10}^{-9}$ | 27105112 |  |
| cg06395091 | chr16:80574926 | *DYNLRB2* | 0.95 | | [0.71,1.27] | 0.74 | 0.82 | HIV infection | | 2.6 | 1·.6${10}^{-16}$ | 27105112 |  |
| cg24654547 | chr16:68057165 | DUS2L;DDX28 | 1.77 | | [1.44,2.19] | 9.29·${10}^{-8}$ | 5.89·${10}^{-7}$ | HIV infection | | -0.88 | 1.4·${10}^{-6}$ | 27105112 |  |
| cg00603498 | chr17:5322775 | RPAIN;NUP88 | 2.45 | | [1.74,3.45] | 2.85·${10}^{-7}$ | 8.26·${10}^{-7}$ | HIV infection | | -0.81 | 9.9·${10}^{-6}$ | 27105112 |  |
| cg24613083 | chr17:59539715 | *TBX4* | 0.73 | | [0.43,1.24] | 0.25 | 0.33 | HIV infection | | -1.40 | 3.9·${10}^{-8}$ | 27105112 |  |
| cg07721276 | chr19:18119064 | *ARRDC2* | 0.96 | | [0.69,1.33] | 0.80 | 0.83 | HIV infection | | 2.3 | 3.3·${10}^{-11}$ | 27105112 |  |
| cg13423282 | chr19:19174731 | *SLC25A42* | 2.62 | | [1.82,3.76] | 1.87·${10}^{-7}$ | 7.00·${10}^{-7}$ | HIV infection | | -0.75 | 1.2·${10}^{-6}$ | 27105112 |  |
| cg01438737 | chr20:42086396 | *SFRS6* | 1.99 | | [1.53,2.6] | 2.91·${10}^{-7}$ | 8.26·${10}^{-7}$ | HIV infection | | -0.63 | 1.2·${10}^{-6}$ | 27105112 |  |
| cg04385144 | chr22:27053419 | *MIAT* | 0.81 | | [0.6,1.11] | 0.19 | 0.27 | Clear cell renal carcinoma | | NA | 8.9·${10}^{-15}$ | 23526956 |  |
| cg01245787 | chr4:71859630 | *DCK* | 2.14 | | [1.54,2.99] | 7.04·${10}^{-6}$ | 1.51·${10}^{-5}$ | Borderline personality disorder vs major depressive disorder | | NA | 3.7·${10}^{-30}$ | 25612291 |  |
| cg21605986 | chr5:175788725 | *KIAA1191* | 2.57 | | [1.85,3.56] | 1.41·${10}^{-8}$ | 2.11·${10}^{-7}$ | HIV infection | | -0.47 | 1.1·${10}^{-6}$ | 27105112 |  |
| cg12995410 | chr6:151712734 | *ZBTB2* | 2.41 | | [1.74,3.34] | 1.37·${10}^{-7}$ | 5.89·${10}^{-7}$ | HIV infection | | -0.39 | 3.8·${10}^{-6}$ | 27105112 |  |
| cg01154537 | chr10:95462167 | *C10orf4* | 1.56 | | [1.25,1.96] | 9.85·${10}^{-5}$ | 1.97·${10}^{-4}$ | HIV infection | | -0.29 | 1.5·${10}^{-5}$ | 27105112 |  |
| cg05557255 | chr11:2422385 | *TSSC4* | 1.81 | | [1.29,2.53] | 5.83·${10}^{-4}$ | 1.03·${10}^{-3}$ | HIV infection | | -0.50 | 2.1·${10}^{-6}$ | 27105112 |  |
| cg16427107 | chr15:75912924 | *SNUPN* | 0.68 | | [0.43,1.08] | 9.97·${10}^{-2}$ | 0.16 | Clear cell renal carcinoma | | NA | 2.3·${10}^{-28}$ | 23526956 |  |
| cg05596135 | chr16:72128010 | *TXNL4B;DHX38* | 1.46 | | [1.95,2.23] | 8.23·${10}^{-2}$ | 0.14 | HIV infection | | 2.3 | 6.3·${10}^{-11}$ | 27105112 |  |

*HR, Hazard Ratio; CI, Confidence Interval of the HR; FDR, False Discovery Rate; PMID, PubMed Identification number; NA, not available*.*

**Table S5. Association of the top CpGs of Factor 27 and cardiovascular disease incidence obtained in the Cox regression analysis adjusted by cell type proportions and one surrogate variable, and annotations among these CpGs and other traits reported in the EWAS catalog.**

| **Feature** | | | **Association with CVD incidence** | | | | **EWAS catalog annotation** | | | | |
| --- | --- | --- | --- | --- | --- | --- | --- | --- | --- | --- | --- |
| *CpG* | *Location* | *Gene* | *HR** | *95% CI** | *P-value* | *FDR correction* | *Trait* | *Beta* | *P-value* | *PMID** |  |
| cg14615927 | chr2:240259213 | *HDAC4* | 1.02 | [0.61,1.72] | 0.94 | 0.94 | Alcohol consumption | 5.8·${10}^{-5}$ | 1.1·${10}^{-10}$ | 27843151 |  |
| cg24408769 | chr6:15506085 | *JARID2* | 2.31 | [1.51,3.55] | 1.18·${10}^{-4}$ | 5.5·${10}^{-4}$ | Age | NA | 1.1·${10}^{-61}$ | 28056824 |  |
| cg03663120 | chr7:2284600 | *NUDT1* | 1.82 | [1.34,2.46] | 1.18·${10}^{-4}$ | 5.5·${10}^{-4}$ | Gestational age | 1·${10}^{-3}$ | 1·${10}^{-9}$ | 27717397 |  |
| cg04290133 | chr7:65439512 | *GUSB* | 1.22 | [0.83,1.81] | 0.31 | 0.34 | Total serum IgE | -6.7 | 7.1·${10}^{-33}$ | 28069425 |  |
| cg13876222 | chr9:139399348 | *NOTCH1* | 2.28 | [1.38,3.77] | 1.37·${10}^{-3}$ | 4.11·${10}^{-3}$ | Alcohol consumption | 6.3·${10}^{-5}$ | 1.6·${10}^{-12}$ | 27843151 |  |
| cg14105458 | chr9:139917436 | *ABCA2* | 1.90 | [1.16,3.10] | 1.05·${10}^{-2}$ | 1.93·${10}^{-2}$ | Fetal vs adult liver | 3.5 | 1.1·${10}^{-27}$ | 25282492 |  |
| cg10967866 | chr10:134362164 | *INPP5A* | 1.36 | [0.85,2.20] | 0.20 | 0.23 | Clear cell renal carcinoma | NA | 9.3·${10}^{-64}$ | 23526956 |  |
| cg19137806 | chr10:134362170 | *INPP5A* | 1.72 | [0.96,3.09] | 6.83·${10}^{-2}$ | 8.90·${10}^{-2}$ | Clear cell renal carcinoma | NA | 2·${10}^{-61}$ | 23526956 |  |
| cg09636302 | chr12:96389483 | *HAL* | 1.71 | [1.14,2.57] | 0.01 | 1.93·${10}^{-2}$ | Fetal vs adult liver | 2.6 | 9.6·${10}^{-41}$ | 25282492 |  |
| cg13645530 | chr12:116756948 | *-* | 2.04 | [1.27,3.29] | 3.36·${10}^{-3}$ | 7.75·${10}^{-3}$ | Clear cell renal carcinoma | NA | 1.6·${10}^{-52}$ | 23526956 |  |
| cg02003183 | chr14:103415882 | *CDC42BPB* | 2.25 | [1.54,3.28] | 2.46·${10}^{-5}$ | 3.69·${10}^{-4}$ | Clear cell renal carcinoma | NA | 4.5·${10}^{-22}$ | 23526956 |  |
| cg11183227 | chr15:91455407 | *MAN2A2* | 2.52 | [1.66,3.81] | 1.35·${10}^{-5}$ | 3.69·${10}^{-4}$ | Body mass index | NA | 2.5·${10}^{-11}$ | 28002404 |  |
| cg00033551 | chr16:4738568 | *MGRN1* | 1.97 | [1.29,3.02] | 1.88·${10}^{-3}$ | 4.69·${10}^{-3}$ | Fetal vs adult liver | 4.6 | 2.9·${10}^{-72}$ | 25282492 |  |
| cg00711896 | chr16:30410051 | *ZNF48* | 1.41 | [0.99,2.01] | 5.51·${10}^{-2}$ | 7.52·${10}^{-2}$ | Age | NA | 9.4·${10}^{-65}$ | 28056824 |  |
| cg03169557 | chr16:89598950 | *SPG7* | 1.59 | [1.14,2.22] | 6.46·${10}^{-3}$ | 1.38·${10}^{-2}$ | Burden of neuritic amyloid plaques | 4.9 | 4·${10}^{-10}$ | 25129075 |  |
| cg03497652 | chr16:4751569 | *ANKS3* | 2.39 | [1.53,3.73] | 1.28·${10}^{-4}$ | 5.5·${10}^{-4}$ | Fetal vs adult liver | 2.6 | 1.1·${10}^{-35}$ | 25282492 |  |
| cg08796240 | chr16:70733832 | VAC14 | 1.88 | [1.36,2.61] | 1.28·${10}^{-4}$ | 5.5·${10}^{-4}$ | Sex | NA | 2.7·${10}^{-5}$ | 26500701 |  |
| cg10505257 | chr16:4731639 | MGRN1 | 1.75 | [1.00,3.04] | 4.83·${10}^{-2}$ | 7.18·${10}^{-2}$ | Fetal vs adult liver | 2.6 | 2.2·${10}^{-38}$ | 25282492 |  |
| cg26992566 | chr16:85814166 | COX4NB | 1.60 | [0.90,2.86] | 0.11 | 0.13 | Clear cell renal carcinoma | NA | 2.7·${10}^{-37}$ | 23526956 |  |
| cg12116137 | chr17:1576449 | *PRPF8* | 2.44 | [1.58,3.77] | 5.60·${10}^{-5}$ | 5.5·${10}^{-4}$ | Age | NA | 1.2·${10}^{-44}$ | 28056824 |  |
| cg14343513 | chr17:78753273 | *RPTOR* | 1.51 | [1.04,2.17] | 2.85·${10}^{-2}$ | 4.75·${10}^{-2}$ | Smoking | 5·${10}^{-3}$ | 3.6·${10}^{-5}$ | 27651444 |  |
| cg21369801 | chr17:80202961 | *CSNK1D* | 1.60 | [0.95,2.68] | 7.46·${10}^{-2}$ | 9.32·${10}^{-2}$ | Alcohol consumption | 2·${10}^{-4}$ | 2.5·${10}^{-9}$ | 27843151 |  |
| cg00994936 | chr19:1423902 | *DAZAP1* | 1.98 | [1.30,3.03] | 1.55·${10}^{-3}$ | 4.23·${10}^{-3}$ | Body mass index | NA | 1.4·${10}^{-16}$ | 28002404 |  |
| cg02715602 | chr19:4544446 | *SEMA6B* | 1.48 | [1.19,1.83] | 3.70·${10}^{-4}$ | 1.39·${10}^{-3}$ | Alcohol consumption | 1.8·${10}^{-4}$ | 2.9·${10}^{-4}$ | 27843151 |  |
| cg04848343 | chr19:4544095 | *SEMA6B* | 2.21 | [1.37,3.55] | 1.16·${10}^{-3}$ | 3.89·${10}^{-3}$ | Gestational age | 3.94·${10}^{-4}$ | 3.2·${10}^{-3}$ | 27717397 |  |
| cg06633438 | chr19:6272158 | *MLLT1* | 1.56 | [1.00,2.43] | 4.93·${10}^{-3}$ | 7.18·${10}^{-2}$ | Clear cell renal carcinoma | NA | 1.1·${10}^{-22}$ | 23526956 |  |
| cg16743273 | chr19:2076833 | *MOBKL2A* | 1.66 | [1.00,2.76] | 0.05 | 7.18·${10}^{-2}$ | Alcohol consumption | 4.6·${10}^{-5}$ | 2.5·${10}^{-12}$ | 27843151 |  |
| cg26325791 | chr19:18234711 | *MAST3* | 1.06 | [0.87,1.29] | 0.56 | 0.58 | Smoking | 7.4·${10}^{-3}$ | 9.9·${10}^{-7}$ | 27651444 |  |
| cg26775538 | chr19:815090 | *LPPR3* | 1.25 | [0.74,2.10] | 0.41 | 0.43 | Age | NA | 3.5·${10}^{-99}$ | 28056824 |  |
| cg27246571 | chr12:96389588 | *HAL* | 1.59 | [1.11,2.28] | 0.01 | 1.93·${10}^{-2}$ | Fetal vs adult liver | 3.1 | 5.5·${10}^{-52}$ | 25282492 |  |

*HR, Hazard Ratio; CI, Confidence Interval of the HR; FDR, False Discovery Rate; PMID, PubMed Identification number; NA, not available*.*

**Table S6.** Association of the MOFA factors and coronary heart disease risk (Cox regression): Model 1, adjusted for cellular types and one surrogate variable; Model 2, additionally adjusted for age and sex; Model 3, additionally adjusted for total cholesterol, HDL-C levels, glucose, smoking status, and systolic and diastolic blood pressure. Factor 21 was stratified by sex, as the interaction between this factor and sex was statistically significant for CVD.

| **Model** | **Association with CHD incidence** | | | |
| --- | --- | --- | --- | --- |
|  | HR* [95% CI*] | P-value | | FDR correction |
| F9* - Model 1 | 1.84 [1.36,2.50] | 8.92·10^-5^ | 1.38·10^-3^ | |
| F9 - Model 2 | 1.65 [1.18,2.30] | 3.44·10^-3^ | 3.21·10^-2^ | |
| F9 - Model 3 | 1.54 [1.09,2.19] | 1.42·10^-2^ | 7.02·10^-2^ | |
| F19* - Model 1 | 1.10 [0.89,1.37] | 0.35 | 0.58 | |
| F19 - Model 2 | 1.02 [0.80,1.29] | 0.86 | 0.95 | |
| F19 - Model 3 | 1.04 [0.82,1.32] | 0.74 | 0.99 | |
| F21* M* - Model 1 | 1.31 [0.96, 1.79] | 8.46·10^-2^ | 0.22 | |
| F21 M - Model 2 | 1.31 [0.96, 1.78] | 9.05·10^-2^ | 0.26 | |
| F21 M - Model 3 | 1.25 [0.92, 1.71] | 0.15 | 0.39 | |
| F21 W* - Model 1 | 1.68 [1.17, 2.42] | 5.41·10^-3^ | 3.36·10^-2^ | |
| F21 W - Model 2 | 1.64 [1.14, 2.36] | 7.62·10^-3^ | 4.72·10^-2^ | |
| F21 W - Model 3 | 1.74 [1.19, 2.53] | 4.06·10^-3^ | 4.73·10^-2^ | |
| F27* - Model 1 | 1.29 [1.09, 1.53] | 3.73·10^-3^ | 2.89·10^-2^ | |
| F27 - Model 2 | 1.31 [1.10, 1.55] | 2.32·10^-3^ | 3.21·10^-2^ | |
| F27 - Model 3 | 1.27 [1.06, 1.52] | 9.56·10^-3^ | 6.24·10^-2^ | |

*CHD, Coronary heart disease; HR, Hazard ratio; CI, Confidence interval; F9, Factor 9; F19, Factor 19; F21, Factor 21; F27, Factor 27; M, men; W, women

**Table S7. Replication of the results in an independent study.** Association of 27 of the top 30 CpGs of Factor 9 with myocardial infarction, assessed by logistic regression in an independent case-control study (REGICOR).

| **Feature** | **Chr*** | **Position*** | **Gene** | **Coef*** | **SE*** | **p-value** | **FDR correction** |
| --- | --- | --- | --- | --- | --- | --- | --- |
| cg03031660 | 17 | 73257791 | *MRPS7;GGA3* | -0.30 | 0.14 | 0.03 | 0.83 |
| cg21028463 | 17 | 74733682 | *MIR636;SFRS2;MFSD11* | -0.34 | 0.17 | 0.05 | 0.86 |
| cg07786668 | 16 | 73092391 | *ZFHX3* | -0.20 | 0.13 | 0.12 | 0.93 |
| cg24341498 | 9 | 137217390 | *RXRA* | -0.20 | 0.16 | 0.21 | 0.96 |
| cg17662034 | 8 | 74207518 | *RDH10* | -0.15 | 0.13 | 0.25 | 0.97 |
| cg24475210 | 4 | 6642433 | *MRFAP1* | 0.12 | 0.14 | 0.41 | 0.98 |
| ch.8.20603847F | 8 | 20559567 | *-* | -0.14 | 0.17 | 0.42 | 0.98 |
| cg19233923 | 11 | 63753598 | *OTUB1* | 0.14 | 0.18 | 0.43 | 0.98 |
| cg11877270 | 2 | 65658583 | *SPRED2* | -0.12 | 0.16 | 0.45 | 0.99 |
| cg24654547 | 16 | 68057165 | *DUS2L;DDX28* | -0.09 | 0.14 | 0.49 | 0.99 |
| cg01438737 | 20 | 42086396 | *SFRS6* | -0.09 | 0.13 | 0.50 | 0.99 |
| cg06642177 | 6 | 134496341 | *SGK1* | -0.09 | 0.13 | 0.50 | 0.99 |
| cg25294185 | 11 | 65487814 | *RNASEH2C* | -0.09 | 0.13 | 0.50 | 0.99 |
| cg22704520 | 2 | 200820451 | *C2orf60;C2orf47* | -0.09 | 0.13 | 0.52 | 0.99 |
| cg19034708 | 8 | 17780168 | *PCM1* | -0.08 | 0.14 | 0.55 | 0.99 |
| cg14037413 | 11 | 9482594 | *ZNF143* | 0.09 | 0.16 | 0.57 | 0.99 |
| cg26260369 | 14 | 68141723 | *VTI1B* | -0.08 | 0.13 | 0.57 | 0.99 |
| cg09238957 | 16 | 46723420 | *ORC6L;VPS35* | 0.07 | 0.14 | 0.59 | 0.99 |
| cg04553410 | 7 | 150864885 | *GBX1* | 0.07 | 0.14 | 0.62 | 0.99 |
| cg26649251 | 19 | 44598564 | *ZNF224;ZNF224* | -0.06 | 0.14 | 0.68 | 0.99 |
| cg05300158 | 4 | 140477727 | *SETD7* | -0.04 | 0.13 | 0.73 | 0.99 |
| cg13390975 | 5 | 34915890 | *BRIX1;RAD1* | -0.04 | 0.13 | 0.75 | 0.99 |
| cg14036868 | 2 | 38604442 | *ATL2* | -0.03 | 0.13 | 0.82 | 1.00 |
| cg18036763 | 22 | 45404910 | *PHF21B* | -0.03 | 0.14 | 0.82 | 1.00 |
| cg22223655 | 18 | 67872902 | *RTTN* | -0.03 | 0.14 | 0.82 | 1.00 |
| cg08965527 | 16 | 84178213 | *HSDL1;LRRC50* | 0.02 | 0.19 | 0.90 | 1.00 |
| cg16120422 | 12 | 113590924 | *CCDC42B* | -0.01 | 0.13 | 0.91 | 1.00 |

*Chr, chromosome; Position, genomic position; Coef, coefficient of the association; SE, standard error of the coefficient; FDR, False Discovery Rate

**Table S8. Replication of the results in an independent study.** Association of 27 of the top 30 CpGs of Factor 19 with myocardial infarction, assessed by logistic regression in an independent case-control study (REGICOR).

| **Feature** | **Chr*** | **Position*** | **Gene** | **Coef*** | **SE*** | **p-value** | **FDR correction** |
| --- | --- | --- | --- | --- | --- | --- | --- |
| cg01127300 | 22 | 38614796 | *-* | -0.41 | 0.15 | 8.59·${10}^{-3}$ | 0.67 |
| cg02513379 | 16 | 27414281 | *IL21R* | 0.37 | 0.15 | 1.68·${10}^{-2}$ | 0.75 |
| cg09479241 | 17 | 27052676 | *TLCD1* | -0.27 | 0.13 | 4.24·${10}^{-2}$ | 0.85 |
| cg01735357 | 20 | 61732467 | *HAR1B;HAR1A* | 0.27 | 0.14 | 5.57·${10}^{-2}$ | 0.88 |
| cg26963277 | 11 | 2722407 | *KCNQ1OT1* | -0.25 | 0.14 | 7.82 ·${10}^{-2}$ | 0.91 |
| cg24146100 | 13 | 99737448 | *DOCK9* | 0.22 | 0.13 | 8.19·${10}^{-2}$ | 0.91 |
| cg25293806 | 19 | 55889387 | *TMEM190* | -0.21 | 0.13 | 9.08·${10}^{-2}$ | 0.92 |
| cg01028796 | 9 | 95525261 | *BICD2* | 0.21 | 0.16 | 0.18 | 0.95 |
| cg04164838 | 19 | 14591148 | *GIPC1* | -0.15 | 0.13 | 0.25 | 0.97 |
| cg06693983 | 19 | 55889216 | *TMEM190* | -0.14 | 0.13 | 0.28 | 0.97 |
| cg07839457 | 16 | 57023022 | *NLRC5* | -0.16 | 0.15 | 0.28 | 0.97 |
| cg00084338 | 6 | 170595920 | *DLL1* | 0.13 | 0.13 | 0.30 | 0.97 |
| cg23363754 | 7 | 29606082 | *PRR15* | -0.13 | 0.13 | 0.31 | 0.97 |
| cg01695406 | 19 | 55889276 | *TMEM190* | -0.14 | 0.14 | 0.32 | 0.97 |
| cg09423312 | 7 | 1163549 | *C7orf50* | -0.13 | 0.14 | 0.34 | 0.98 |
| cg03494429 | 12 | 4417093 | *-* | 0.13 | 0.14 | 0.35 | 0.99 |
| cg06868100 | 7 | 29606349 | *PRR15* | -0.07 | 0.13 | 0.58 | 0.99 |
| cg08952306 | 7 | 101962123 | *SH2B2* | 0.07 | 0.14 | 0.56 | 0.99 |
| cg14281592 | 9 | 129884313 | *ANGPTL2;RALGPS1* | 0.075 | 0.15 | 0.61 | 0.99 |
| cg14170999 | 17 | 79380515 | *BAHCC1* | 0.06 | 0.12 | 0.62 | 0.99 |
| cg10064322 | 19 | 14591203 | *GIPC1* | -0.06 | 0.15 | 0.70 | 0.99 |
| cg22878489 | 6 | 33245701 | *B3GALT4* | -0.05 | 0.13 | 0.71 | 0.99 |
| cg19492423 | 19 | 18700933 | *C19orf60* | 0.03 | 0.13 | 0.82 | 1 |
| cg16737517 | 20 | 62406677 | *ZBTB46* | -0.03 | 0.13 | 0.84 | 1 |
| cg26372517 | 1 | 36039159 | *TFAP2E* | 0.02 | 0.15 | 0.88 | 1 |
| cg11908057 | 7 | 27171154 | *HOXA4* | -0.01 | 0.14 | 0.93 | 1 |
| cg25979108 | 19 | 18700553 | *C19orf60* | 6.95·${10}^{-3}$ | 0.15 | 0.96 | 1 |

*Chr, chromosome; Position, genomic position; Coef, coefficient of the association; SE, standard error of the coefficient; FDR, False Discovery Rate

**Table S9. Replication of the results in an independent study.** Association of 28 of the top 30 CpGs of Factor 21 with myocardial infarction, assessed by logistic regression in an independent case-control study (REGICOR).

| **Feature** | **Chr*** | **Position*** | **Gene** | **Coef*** | **SE*** | **p-value** | **FDR correction** |
| --- | --- | --- | --- | --- | --- | --- | --- |
| cg04364194 | 1 | 202114085 | *ARL8A* | -0.31 | 0.14 | 2.74·${10}^{-2}$ | 0.81 |
| cg24609304 | 2 | 32483287 | *NLRC4* | -0.22 | 0.13 | 9.55·${10}^{-2}$ | 0.92 |
| cg16427107 | 15 | 75912924 | *SNUPN* | 0.28 | 0.17 | 9.92·${10}^{-2}$ | 0.93 |
| cg07721276 | 19 | 18119064 | *ARRDC2* | -0.23 | 0.15 | 0.12 | 0.93 |
| cg07790826 | 11 | 70049435 | *FADD* | -0.12 | 0.13 | 0.14 | 0.94 |
| cg06750635 | 11 | 108093419 | *ATM;NPAT* | -0.16 | 0.14 | 0.28 | 0.97 |
| cg10041390 | 10 | 89623018 | *PTEN;KILLIN* | -0.11 | 0.13 | 0.41 | 0.98 |
| cg01245787 | 4 | 71859630 | *DCK* | -0.14 | 0.17 | 0.41 | 0.98 |
| cg00603498 | 17 | 5322775 | *RPAIN;NUP88* | -0.10 | 0.14 | 0.46 | 0.99 |
| cg24654547 | 16 | 68057165 | *DUS2L;DDX28* | -0.09 | 0.14 | 0.49 | 0.99 |
| cg01438737 | 20 | 42086396 | *SFRS6* | -0.09 | 0.13 | 0.50 | 0.99 |
| cg06642177 | 6 | 134496341 | *SGK1* | -0.09 | 0.13 | 0.50 | 0.99 |
| cg07959491 | 1 | 204347150 | *-* | -0.09 | 0.14 | 0.52 | 0.99 |
| cg06395091 | 16 | 80574926 | *DYNLRB2* | -0.08 | 0.14 | 0.56 | 0.99 |
| cg10341991 | 3 | 48229869 | *CDC25A* | -0.08 | 0.14 | 0.57 | 0.99 |
| cg04385144 | 22 | 27053419 | *MIAT* | -0.07 | 0.13 | 0.61 | 0.99 |
| cg02994863 | 1 | 64059297 | *PGM1* | -0.06 | 0.13 | 0.63 | 0.99 |
| cg05596135 | 16 | 72128010 | *TXNL4B;DHX38* | -0.05 | 0.17 | 0.75 | 0.99 |
| cg04509559 | 14 | 69864994 | *ERH;SLC39A9* | 0.04 | 0.14 | 0.80 | 1 |
| cg15062310 | 7 | 100450120 | *SLC12A9* | -0.03 | 0.13 | 0.81 | 1 |
| cg21605986 | 5 | 175788725 | *KIAA1191* | -0.03 | 0.17 | 0.85 | 1 |
| cg25183890 | 8 | 94929275 | *PDP* | -0.02 | 0.13 | 0.85 | 1 |
| cg24613083 | 17 | 59539715 | *TBX4* | -0.02 | 0.14 | 0.88 | 1 |
| cg13423282 | 19 | 19174731 | *SLC25A42* | -0.02 | 0.13 | 0.90 | 1 |
| cg26258108 | 2 | 232329189 | *NCL* | -5.91·${10}^{-3}$ | 0.13 | 0.96 | 1 |
| cg01154537 | 10 | 95462167 | *C10orf4* | -5·${10}^{-3}$ | 0.18 | 0.98 | 1 |
| cg14860120 | 15 | 48470606 | *MYEF2* | 3.8·${10}^{-3}$ | 0.14 | 0.98 | 1 |

*Chr, chromosome; Position, genomic position; Coef, coefficient of the association; SE, standard error of the coefficient; FDR, False Discovery Rate

**Table S10. Replication of the results in an independent study.** Association of the top 30 CpGs of Factor 27 with myocardial infarction, assessed by logistic regression in an independent case-control study (REGICOR).

| **Feature** | **Chr*** | **Position*** | **Gene** | **Coef*** | **SE*** | **p-value** | **FDR correction** |
| --- | --- | --- | --- | --- | --- | --- | --- |
| cg02003183 | 14 | 103415882 | *CDC42BPB* | 0.50 | 0.14 | 3·${10}^{-4}$ | 0.27 |
| cg00711896 | 16 | 30410051 | *ZNF48* | 0.44 | 0.14 | 2.40·${10}^{-3}$ | 0.49 |
| cg11183227 | 15 | 91455407 | *MAN2A2* | 0.43 | 0.17 | 1.01·${10}^{-2}$ | 0.69 |
| cg13645530 | 12 | 116756948 | *-* | 0.29 | 0.16 | 7.04·${10}^{-2}$ | 0.90 |
| cg06633438 | 19 | 6272158 | *MLLT1* | -0.30 | 0.18 | 9.35·${10}^{-2}$ | 0.92 |
| cg26992566 | 16 | 85814166 | *COX4NB* | -0.25 | 0.17 | 0.14 | 0.94 |
| cg04848343 | 19 | 4544095 | *SEMA6B* | -0.16 | 0.14 | 0.25 | 0.97 |
| cg26325791 | 19 | 18234711 | *MAST3* | 0.15 | 0.13 | 0.27 | 0.97 |
| cg03663120 | 7 | 2284600 | *NUDT1* | 0.16 | 0.14 | 0.28 | 0.97 |
| cg19137806 | 10 | 134362170 | *INPP5A* | 0.16 | 0.14 | 0.27 | 0.97 |
| cg04290133 | 7 | 65439512 | *GUSB* | 0.15 | 0.14 | 0.28 | 0.97 |
| cg14105458 | 9 | 139917436 | *ABCA2* | -0.16 | 0.15 | 0.29 | 0.97 |
| cg10967866 | 10 | 134362164 | *INPP5A* | 0.15 | 0.15 | 0.30 | 0.97 |
| cg00994936 | 19 | 1423902 | *DAZAP1* | 0.14 | 0.15 | 0.35 | 0.98 |
| cg08796240 | 16 | 70733832 | *VAC14* | -0.13 | 0.14 | 0.38 | 0.98 |
| cg09636302 | 12 | 96389483 | *HAL* | -0.13 | 0.15 | 0.39 | 0.98 |
| cg00033551 | 16 | 4738568 | *MGRN1* | 0.10 | 0.14 | 0.47 | 0.99 |
| cg14343513 | 17 | 78753273 | *RPTOR* | 0.11 | 0.16 | 0.49 | 0.99 |
| cg13876222 | 9 | 139399348 | *NOTCH1* | 0.10 | 0.15 | 0.50 | 0.99 |
| cg03497652 | 16 | 4751569 | *ANKS3* | 0.10 | 0.14 | 0.52 | 0.99 |
| cg26775538 | 19 | 815090 | *LPPR3* | 0.10 | 0.16 | 0.55 | 0.99 |
| cg21369801 | 17 | 80202961 | *CSNK1D* | -7.65·${10}^{-2}$ | 0.17 | 0.65 | 0.99 |
| cg24408769 | 6 | 15506085 | *JARID2* | -6.64·${10}^{-2}$ | 0.14 | 0.66 | 0.99 |
| cg02715602 | 19 | 4544446 | *SEMA6B* | -5.43·${10}^{-2}$ | 0.14 | 0.69 | 0.99 |
| cg27246571 | 12 | 96389588 | *HAL* | -4.84·${10}^{-2}$ | 0.15 | 0.75 | 0.99 |
| cg14615927 | 2 | 240259213 | *HDAC4* | 6.22·${10}^{-2}$ | 0.20 | 0.75 | 0.99 |
| cg03169557 | 16 | 89598950 | *SPG7* | 2.57·${10}^{-2}$ | 0.14 | 0.86 | 1 |

*Chr, chromosome; Position, genomic position; Coef, coefficient of the association; SE, standard error of the coefficient; FDR, False Discovery Rate
