# Supplementary material for: DNA methylation and gene expression integration in cardiovascular disease
Source: Clin Epigenetics. 2021 Apr 9;13:75. doi: 10.1186/s13148-021-01064-y (PMC8034168; doi:10.1186/s13148-021-01064-y)
Supplement: Supplementary file 2 — Additional file 2: Supplementary figures. [file 13148_2021_1064_MOESM2_ESM.docx]

**SUPPLEMENTARY FIGURES**

**
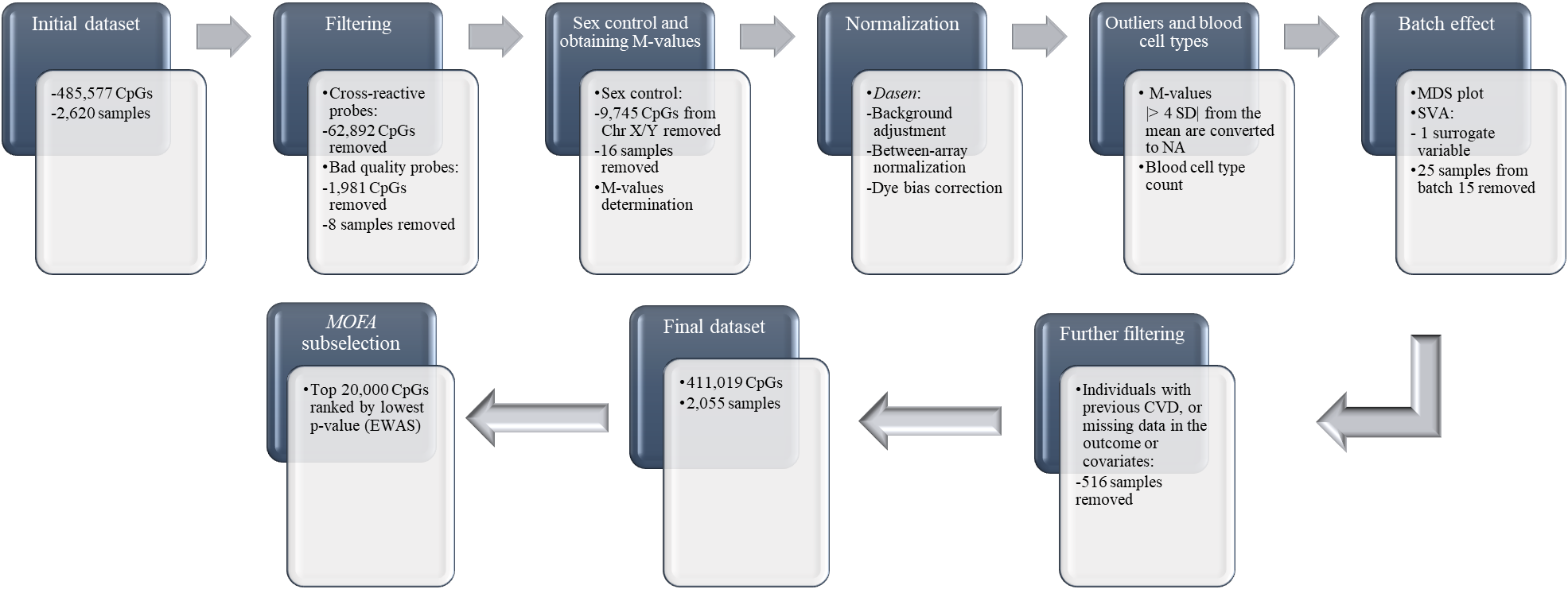
**

**Figure S1.** Flowchart of the quality control steps of the DNA methylation data.


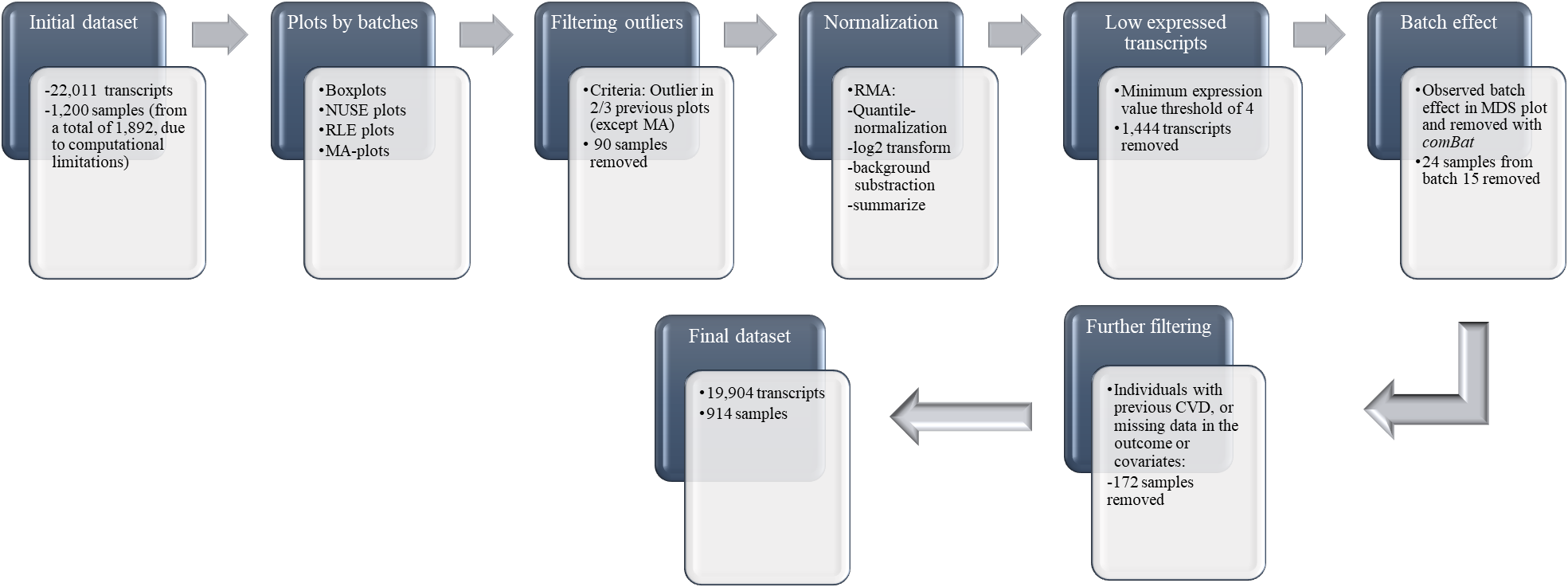


**Figure S2.** Flowchart of the quality control steps of the gene expression data.


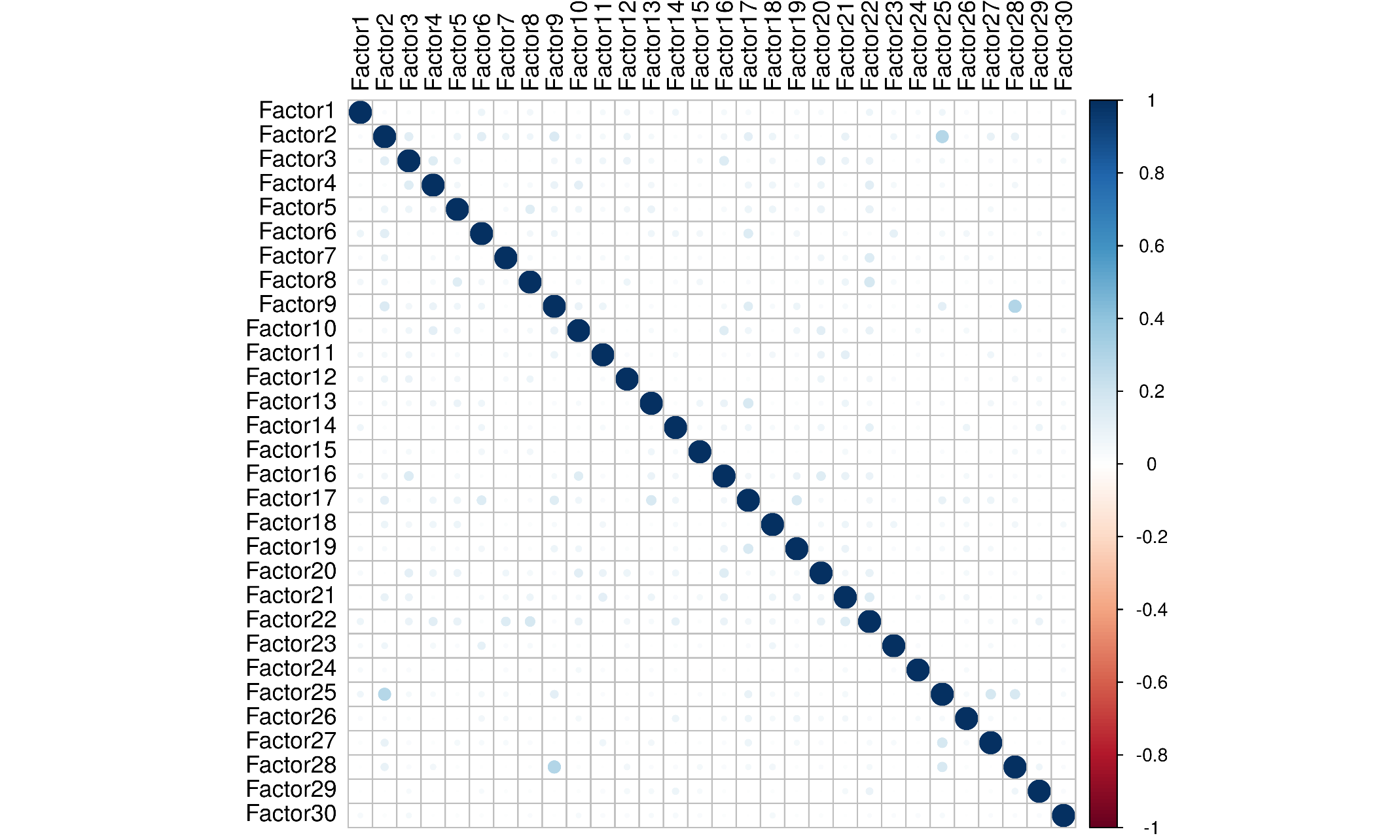


**Figure S3. Correlations between factors.** Correlation coefficients are represented in a colour scale from red tones for negative correlations to blue tones for positive correlations. Values are below 0.20, indicating that factors are capturing largely independent sources of variation in the data set.

**B**

**A**

**
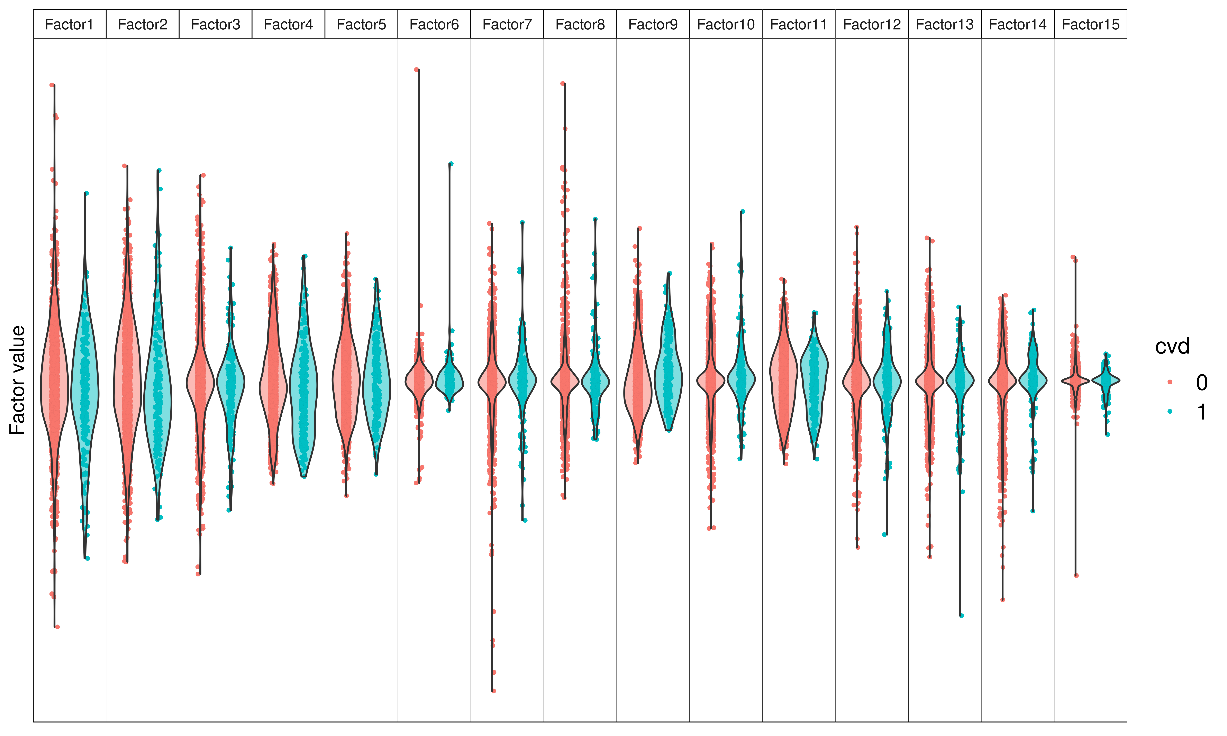

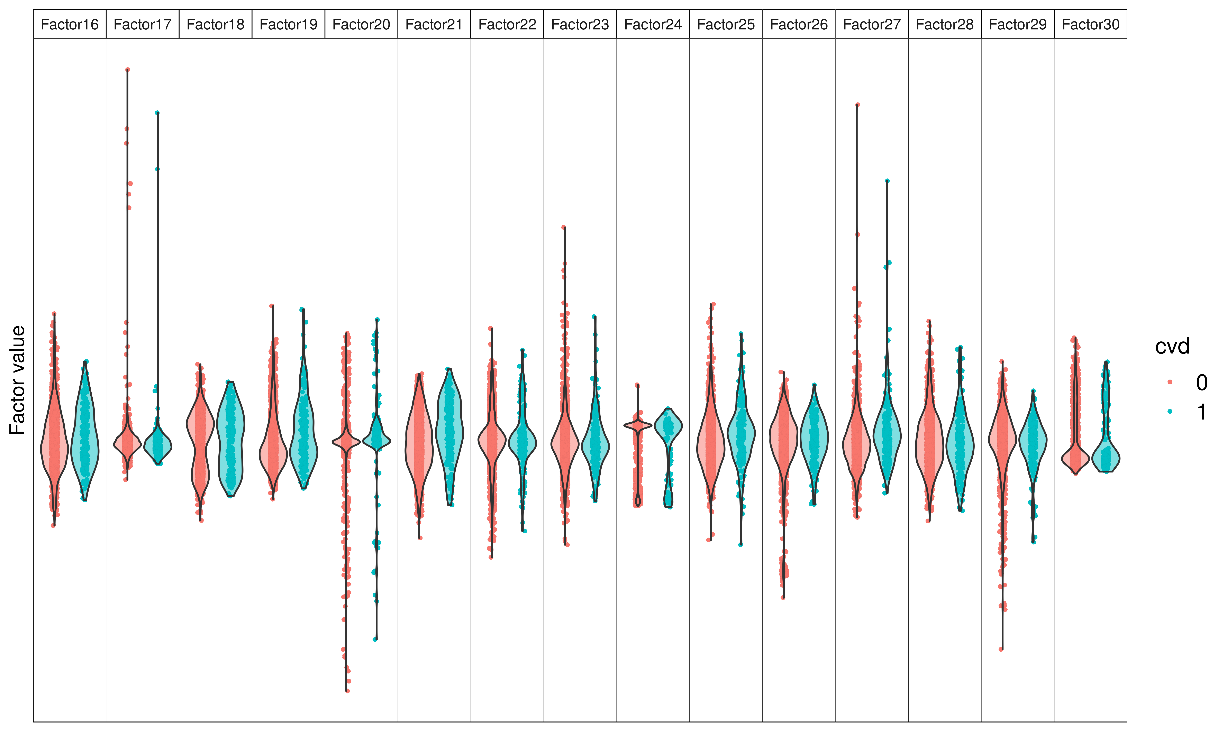
**

**Figure S4. Violin plots of the 30 factors identified by multi-omic factor analysis, stratified according to the presence of cardiovascular disease (red: absence; blue: presence).** (A) factors 1-15, (B) factors 16-30.


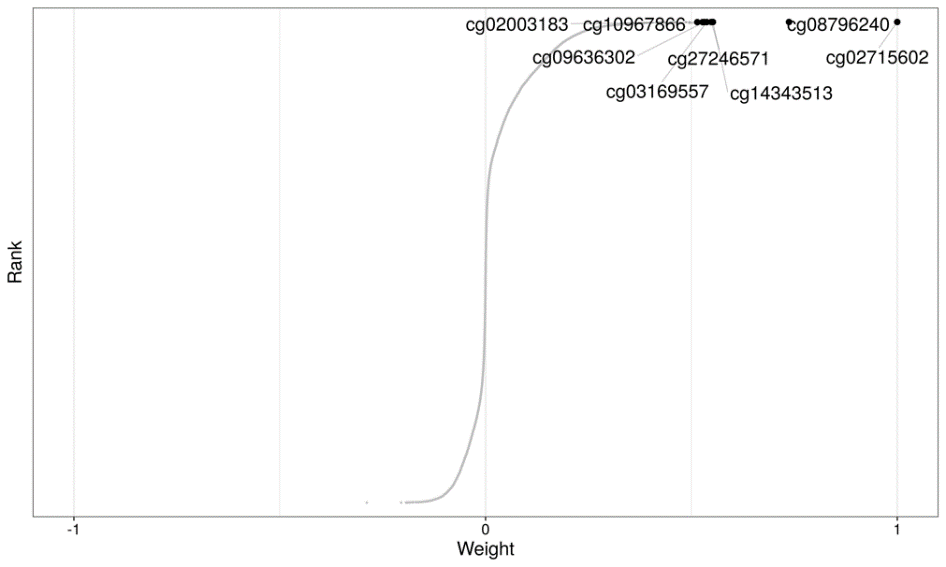

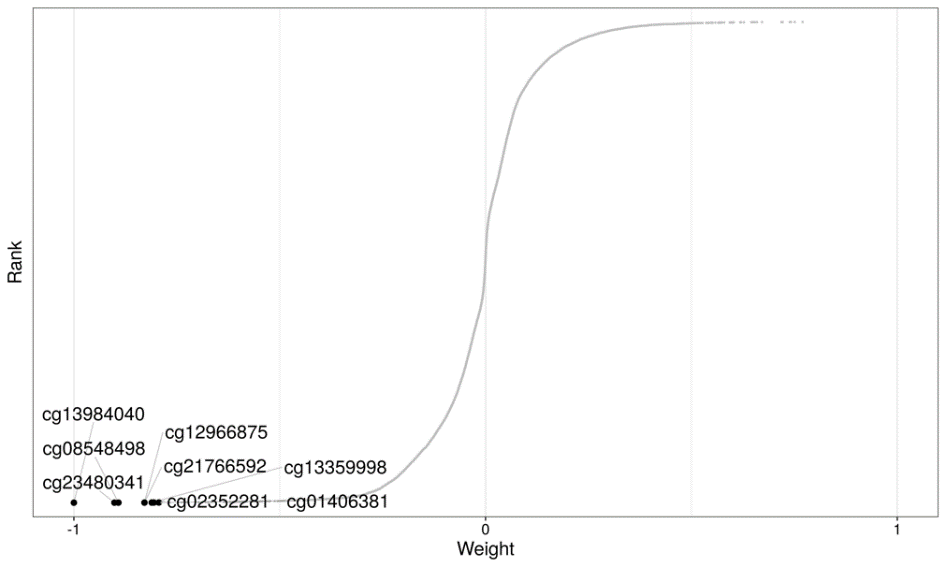

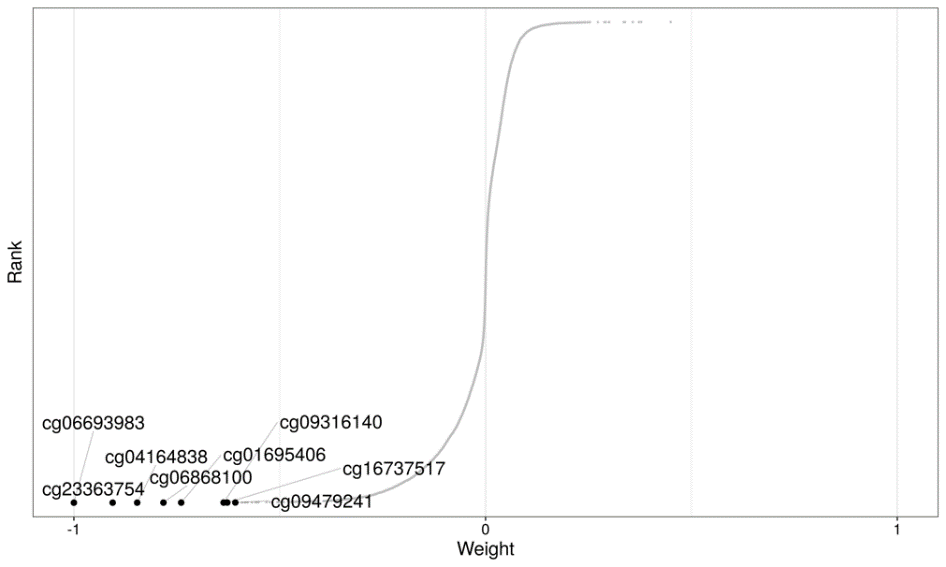

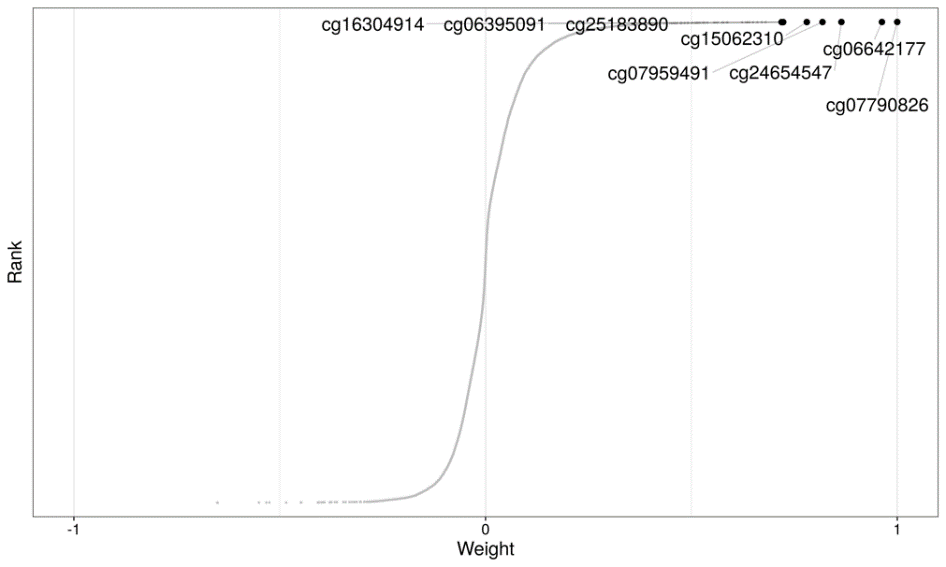


**A**

**B**

**C**

**D**

**Figure S5. Overall features loading weights from factors 9 (A), 19 (B), 21 (C) and 27 (D).** Values range from -1 to 1. Features associated with the factor have larger absolute values, whereas features with no association are expected to have values close to zero. A positive weight indicates higher levels of that feature in samples with positive factor values, and vice versa.


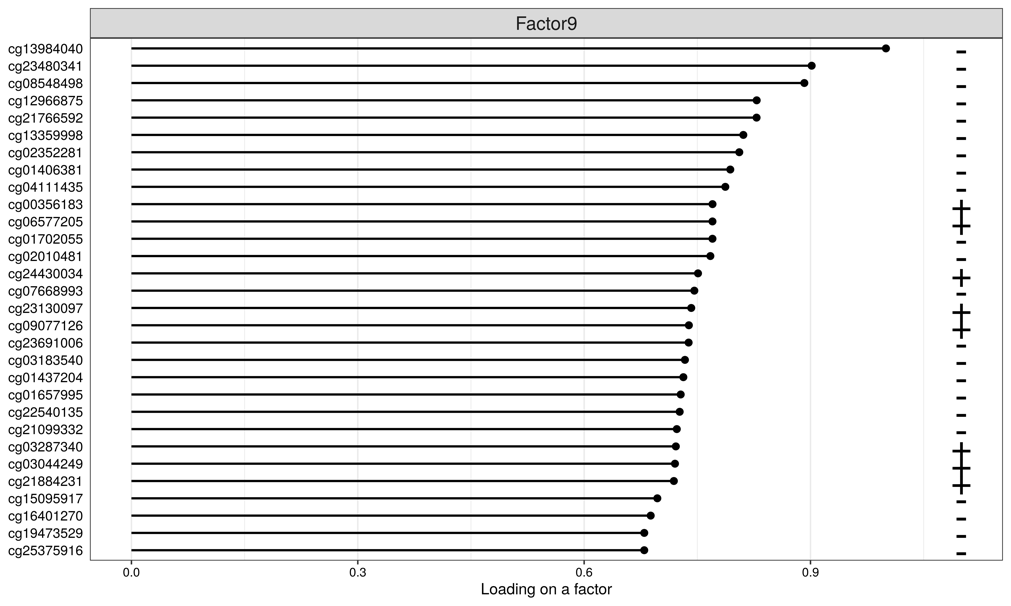

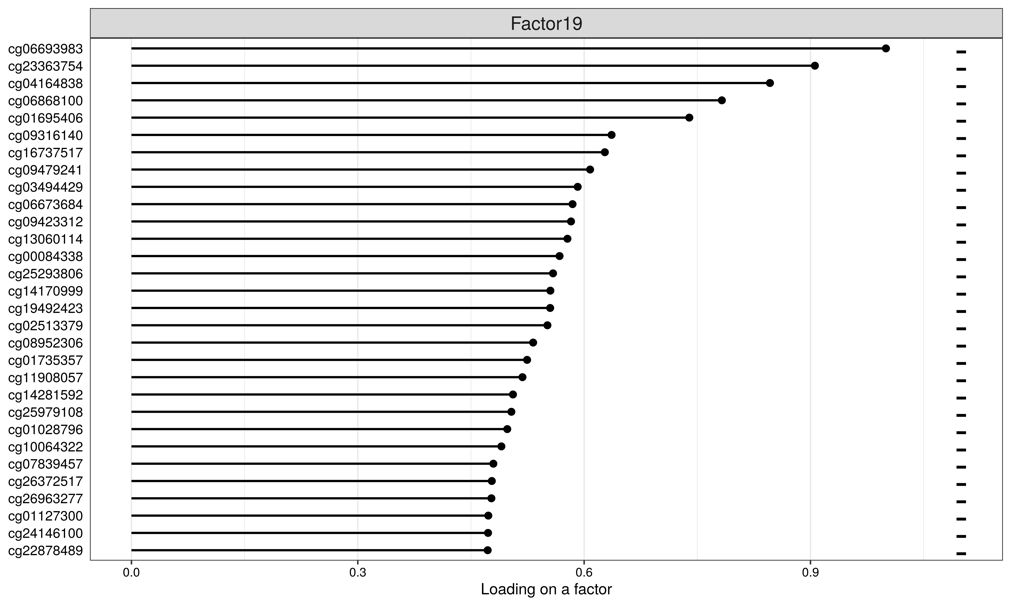

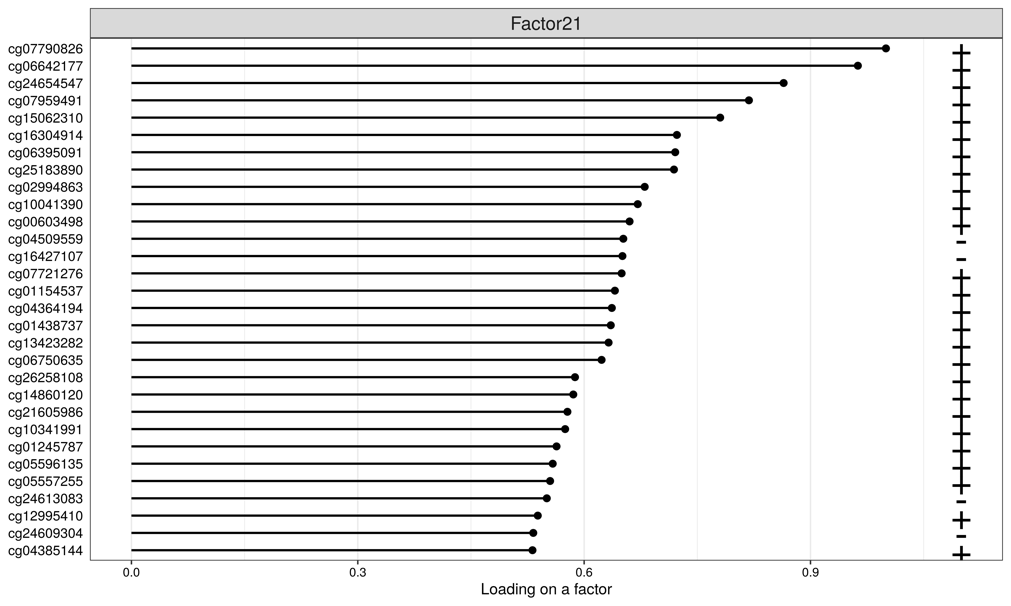

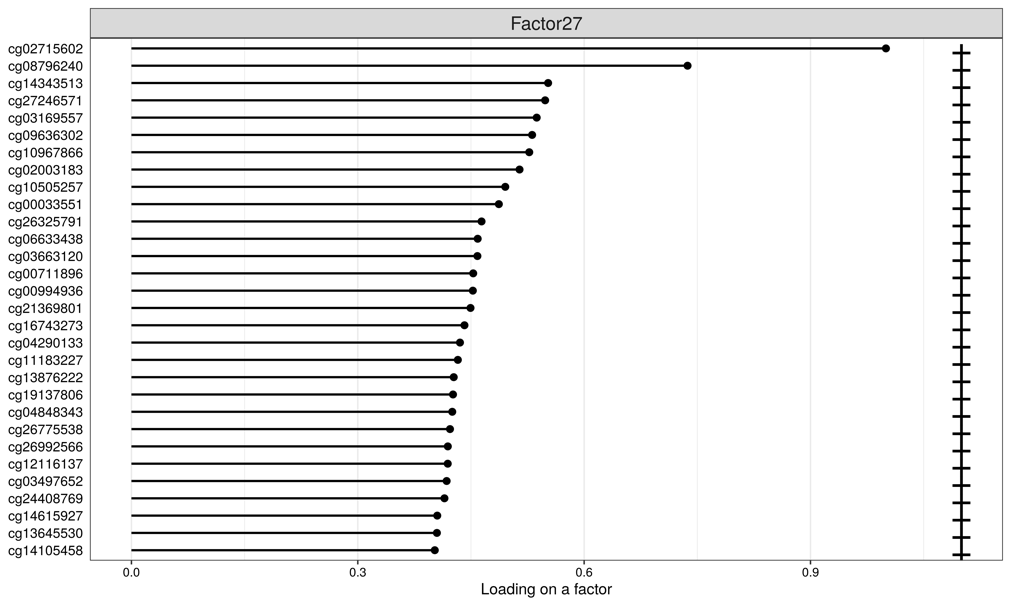


**A**

**B**

**C**

**D**

**Figure S6.** **Top 30** **features (CpGs) loading weights from factors 9 (A) 19 (B), 21 (C) and 27 (D).** Values ranges from 0 to 1 in absolute scale. Features associated with the factor have larger absolute values.


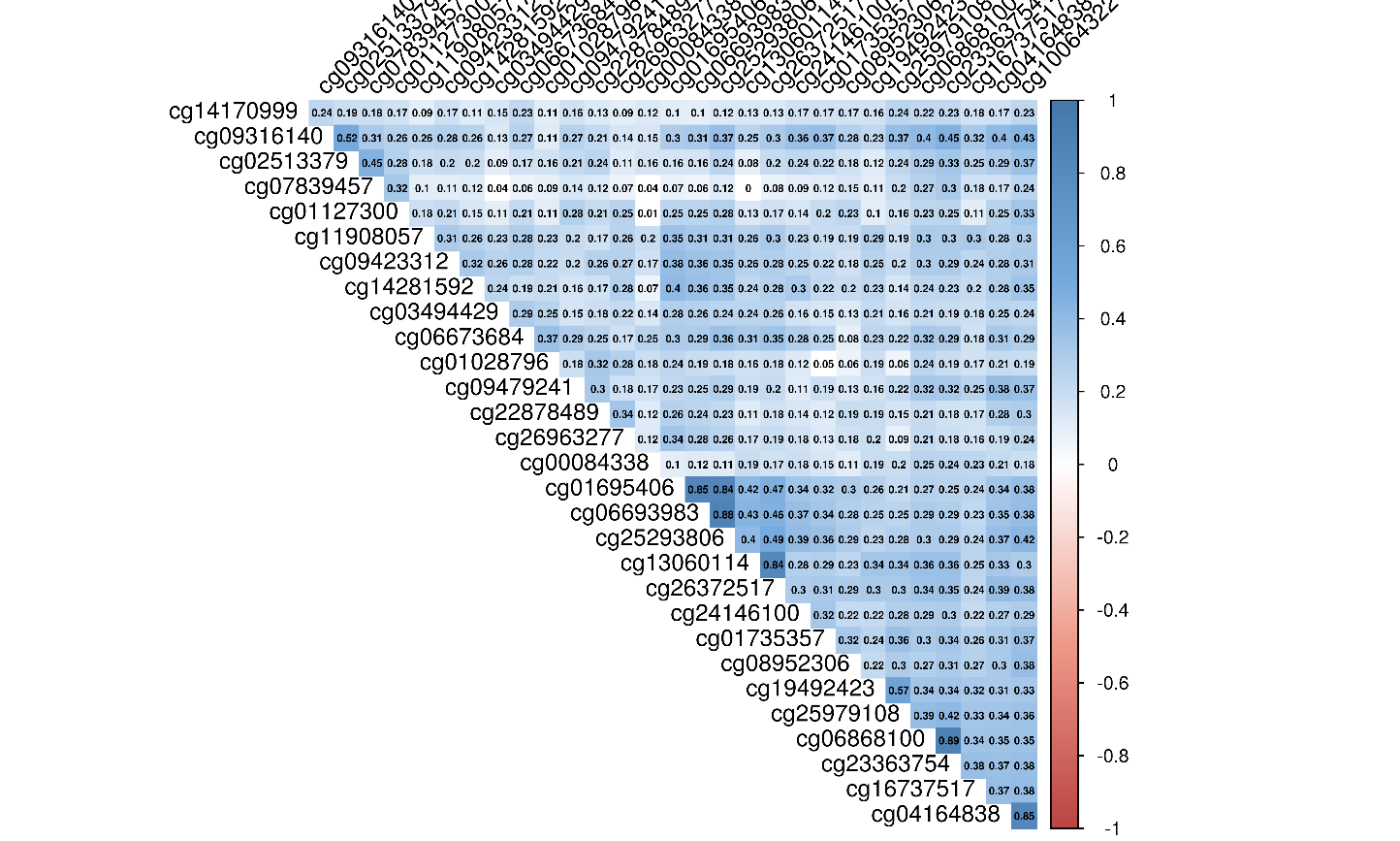

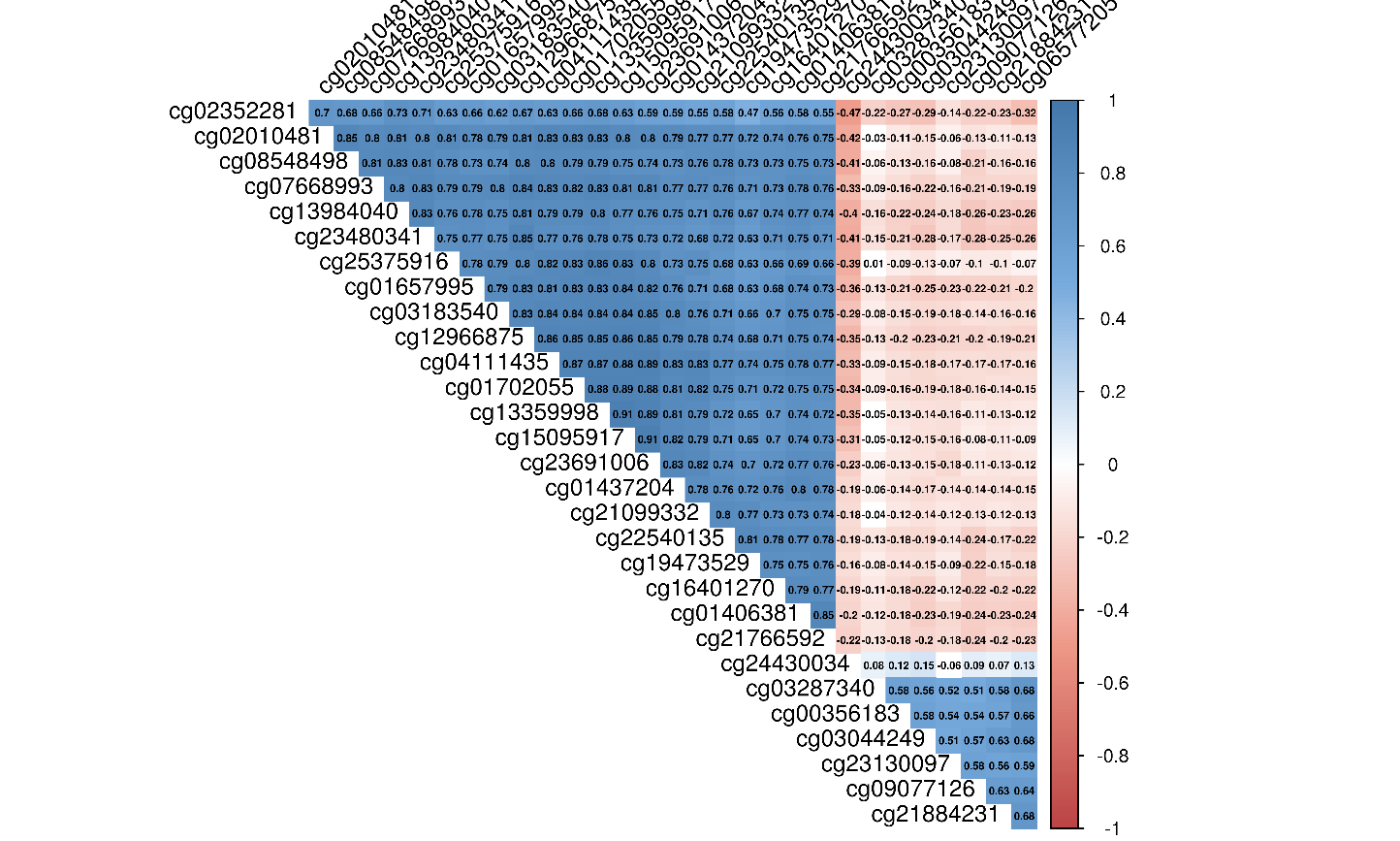


**B**

**A**

**C**

**D**


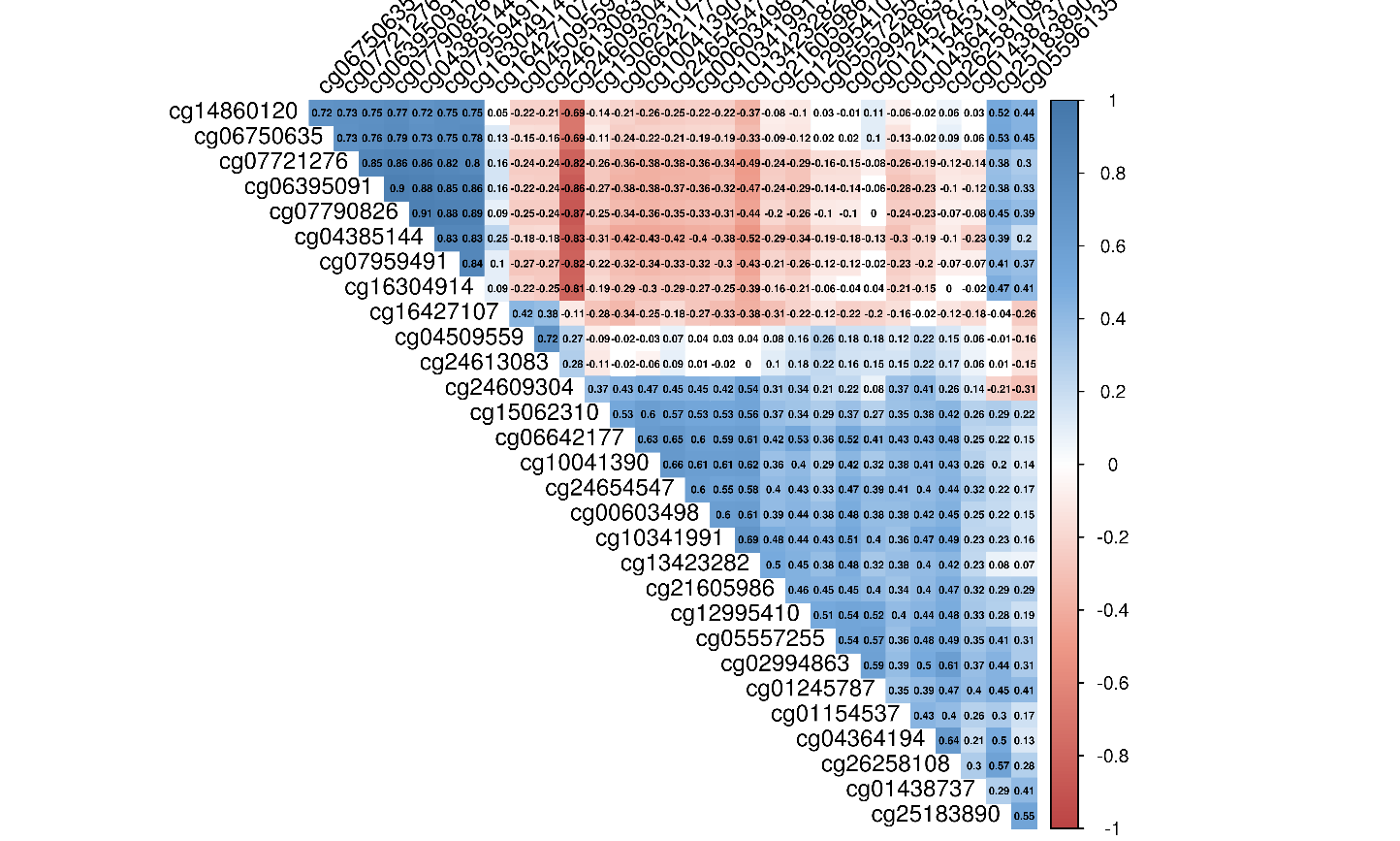

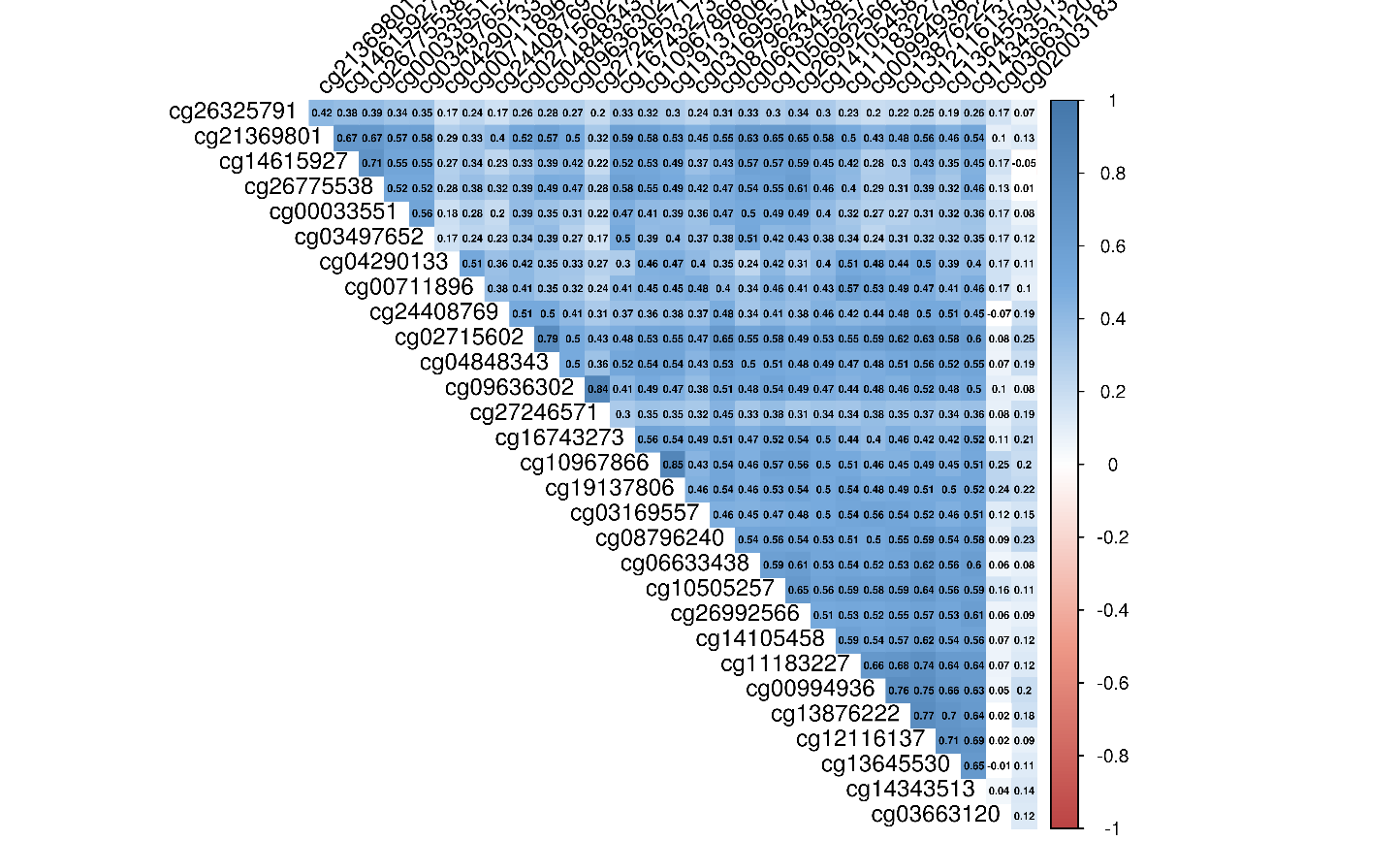


**Figure S7. Correlations coefficients between the top 30 CpGs from factors 9 (A), 19 (B), 21 (C) and 27 (D)**. Correlation coefficients are represented in a color scale from red tones for negative correlations to blue tones for positive correlations.
