# Supplementary material for: DNA methylation and gene expression integration in cardiovascular disease
Source: Clin Epigenetics. 2021 Apr 9;13:75. doi: 10.1186/s13148-021-01064-y (PMC8034168; doi:10.1186/s13148-021-01064-y)
Supplement: Supplementary file 3 — Additional file 3: Supplementary material and methods. [file 13148_2021_1064_MOESM3_ESM.docx]

**SUPPLEMENTARY MATERIAL**

**SUPPLEMENTARY METHODS**

**Replication of the top features from the CVD-related MOFA factors in an independent study**

The 30 CpGs with largest weights in those factors associated with CVD in the Framingham Offspring Study were assessed for replication in an independent study (REGICOR; REgistre GIroní del COR) (Fernández-Sanlés *et al.*, 2019). The study design, DNA methylation and covariates assessment, and the quality control have already been described in detail (Fernández-Sanlés *et al.*, 2019). Here, we describe them briefly. All the analyses were performed using R packages available through the Bioconductor repository (Huber *et al.,* 2015).

*REGICOR population*

REGICOR includes both a population-based myocardial infarction register and a population-based survey in the same geographical area in the province of Girona. We designed an age- and sex-matched case-control study: cases were patients with an acute myocardial infarction consecutively attended in the reference hospital of Girona. The study included 208 myocardial infarction cases (women were overrepresented, 50% of the sample). Controls were randomly selected from a population-based survey performed in the same area in 2009-2013. All participants were of European descent.

*Assessment of DNA methylation status*

The quality of DNA from the participants of the REGICOR case-control study was first checked with Picogreen (Thermo Fisher Scientific, MA, USA), and afterwards analysed in the Genomics and Epigenomics Service of the Bellvitge Institute for Biomedical Research (Barcelona, Spain), distributed in 13 batches of the Infinium MethylationEPIC BeadChip. Methylation status at each CpG site was reported by M-values.

*Quality control of the samples analysed with the Illumina MethylationEPIC BeadChip*

Quality control included the exclusion of samples with a detection p-value > 0.05 in at least 1% of the probes, and of those CpGs with a beadcount < 3 in at least 5% of the samples, using the *wateRmelon* R package (Pidsley *et al.*, 2013). We also excluded cross-reactive probes (n = 43,979), and those reported by Illumina to be discarded due to underperformance (n = 1,031) and changes in the manufacturing process (n = 977). In addition, those samples which show inconsistent methylation-based predicted sex and reported sex were also removed using *methylumi* R package (Davis *et al.*, 2020). Finally, M-values above 4 standard deviations from the average in absolute value were not considered in the subsequent analyses.

*Covariates assessment*

The REGICOR study prospectively collects blood samples, sociodemographic, lifestyle and clinical data using validated methods and questionnaires. Furthermore, *FlowSorted.Blood.450k* R package (Jaffe, 2019) was used to obtain methylation-based estimations of the blood cell type counts (B Cells, Monocytes, Granulocytes, Natural Killers, CD4+ T cells and CD8+ T helper cells). In addition, *sva* R package (Leek *et al.*, 2019) was used to obtain a maximum of two surrogate variables. Both, cell type counts and surrogate variables were used as covariates.

*Statistical analysis*

The top 30 CpGs with largest weights from factors associated with CVD (factors 19, 21, 27) were analyzed. Logistic regression was used, myocardial infarction was treated as outcome or dependent variable and DNA methylation as the exposure or independent variable. The model was adjusted for the estimated cell counts (CD8+ T Cells, CD4+ T cells, Natural Killer cells, B cells, Monocytes, Granulocytes) and two surrogate variables. The study was sex- and age-adjusted by design. We applied FDR to correct for multiple comparisons.

**REFERENCES**

Davis, S. *et al.* (2020) methylumi: Handle Illumina methylation data. R package.

Fernández-Sanlés, A. *et al.* (2019) DNA Methylation Biomarkers Of Myocardial Infarction And Cardiovascular Disease, *bioRxiv*, 3, 707315.

Jaffe, A. E. (2019) FlowSorted.Blood.450k: Illumina HumanMethylation data on sorted blood cell populations. R package.

Leek, J. T. *et al.* (2019) sva: Surrogate Variable Analysis. R package.

Pidsley, R. *et al.* (2013) A data-driven approach to preprocessing Illumina 450K methylation array data’, *BMC Genomics*, 14(1), 293.

Wolfgang Huber *et al.* (2015). Orchestrating high-throughput genomic analysis with Bioconductor. *Nature methods,* 12, 115-121.
